# Physiologic relevance of the transpulmonary metabolome in connective tissue disease–associated pulmonary vascular disease

**DOI:** 10.1172/jci.insight.187911

**Published:** 2025-05-08

**Authors:** Michael H. Lee, Thaís C. F. Menezes, Julie A. Reisz, Francesca I. Cendali, Eloara V. M. Ferreira, Jaquelina S. Ota-Arakaki, Priscila A. Sperandio, Rahul Kumar, Claudia Mickael, Martin M. Ieong, Juliana Lucena Santos, Ana Carolina B. Duarte, Dara C. Fonseca Balladares, Kevin Nolan, Rubin M. Tuder, Paul M. Hassoun, Angelo D’Alessandro, Rudolf K. F. Oliveira, Brian B. Graham

**Affiliations:** 1 Division of Pulmonary and Critical Care Medicine, Department of Medicine, UCSF, San Francisco, California, USA.; 2 Lung Biology Center, Department of Medicine, Zuckerberg San Francisco General Hospital, San Francisco, California, USA.; 3 Division of Respiratory Diseases, Department of Medicine, Federal University of São Paulo (UNIFESP), Hospital São Paulo, São Paulo, Brazil.; 4 Department of Biochemistry and Molecular Genetics, and; 5 Division of Pulmonary Sciences and Critical Care Medicine, Department of Medicine, University of Colorado Anschutz Medical Campus, Aurora, Colorado, USA.; 6 Division of Pulmonary and Critical Care Medicine, Department of Medicine, Johns Hopkins University School of Medicine, Baltimore, Maryland, USA.

**Keywords:** Metabolism, Pulmonology, Vascular biology, Autoimmune diseases, Cardiovascular disease, Rheumatology

## Abstract

Pathologic implications of dysregulated pulmonary vascular metabolism to pulmonary arterial hypertension (PAH) are increasingly recognized, but their clinical applications have been limited. We hypothesized that metabolite quantification across the pulmonary vascular bed in connective tissue disease–associated (CTD-associated) PAH would identify transpulmonary gradients of pathobiologically relevant metabolites, in an exercise stage–specific manner. Sixty-three CTD patients with established or suspected PAH underwent exercise right heart catheterization. Using mass spectrometry–based metabolomics, metabolites were quantified in plasma samples simultaneously collected from the pulmonary and radial arteries at baseline and during resistance-free wheeling, peak exercise, and recovery. We identified uptake and excretion of metabolites across the pulmonary vascular bed, unique and distinct from single vascular site analysis. We demonstrated the physiological relevance of metabolites previously shown to promote disease in animal models and end-stage human lung tissues, including acylcarnitines, glycolytic intermediates, and tryptophan catabolites. Notably, pulmonary vascular metabolite handling was exercise stage specific. Transpulmonary metabolite gradients correlated with hemodynamic endpoints largely during free-wheeling. Glycolytic intermediates demonstrated physiologic significance at peak exercise, including net uptake of lactate in those with more advanced disease. Contribution of pulmonary vascular metabolism to CTD-PAH pathogenesis and therapeutic candidacy of metabolism modulation must be considered in the context of physiologic stress.

## Abstract 2

Connective tissue disease-associated pulmonary vascular disease is characterized by exercise stage-specific gradients of pathogenic metabolites across the pulmonary vascular bed.

## Introduction

Pulmonary arterial hypertension (PAH) is an incurable disease characterized by remodeled pulmonary vasculature, ultimately resulting in right ventricular failure and death (1, 2). Despite growing knowledge of the pathobiology and potential therapeutic targets in PAH, its clinical outcome remains poor (3, 4). This grim prognosis and early fatality are particularly true in a subset of patients whose PAH is caused by connective tissue diseases (CTD-PAH) (5). One promising area of research is the role of metabolic pathways in PAH pathogenesis, as pro-growth, pro-survival effects of metabolic signals are believed to promote pulmonary vascular proliferation (6).

Successful clinical applications of metabolic modulation to PAH management, however, have been limited (7, 8). Underlying the barrier to therapeutically targeting metabolism in PAH is a paucity of information on pulmonary vascular metabolism in living, functional patients with earlier stage PAH. Due to high procedural risks (9), lung biopsies and pulmonary artery isolations have been feasible only in explanted or autopsy tissues from patients with end-stage PAH, leaving the human pulmonary vasculature metabolic profile largely uncharacterized in preterminal stages.

Similarly, little is known about how pulmonary vascular metabolism changes with exercise, the time when patients with PAH are most symptomatic, as most studies are done at rest. In the healthy pulmonary vasculature, exercise produces dilation and recruitment of the pulmonary arteries to accommodate more blood flow, quantified as pulmonary arterial compliance (10), ultimately resulting in reduced pulmonary vascular resistance (PVR) (11, 12). Conversely, the absence of these physiologic adaptations during exercise has been shown to predict worse outcomes in PAH (13–16). Physiologic implications of dysregulated pulmonary vascular metabolism in PAH, however, remain unknown.

Previous studies attempted to deduce metabolic changes in the pulmonary vasculature of patients with PAH by analyzing their plasma metabolome (17–28). The clinical applicability of these findings, however, has been limited by (a) plasma collection from a single anatomic site reflecting metabolic disturbances of the whole body rather than the pulmonary vessels and (b) the incomprehension of how metabolism relates to exercise physiology in PAH. We therefore aimed to quantify the transpulmonary (i.e., across the pulmonary vascular bed) plasma metabolome in exercising CTD patients with suspected or confirmed PAH, with the goal of identifying metabolic signatures specific to the pulmonary vasculature and defining how they relate to physiologic, clinical parameters. Of many subtypes of PAH, we focused on CTD-PAH given its particularly poor outcomes and our recent report of dysregulated pulmonary vascular metabolism in CTD-PAH (29).

## Results

### Study population.

**Figure 1:**
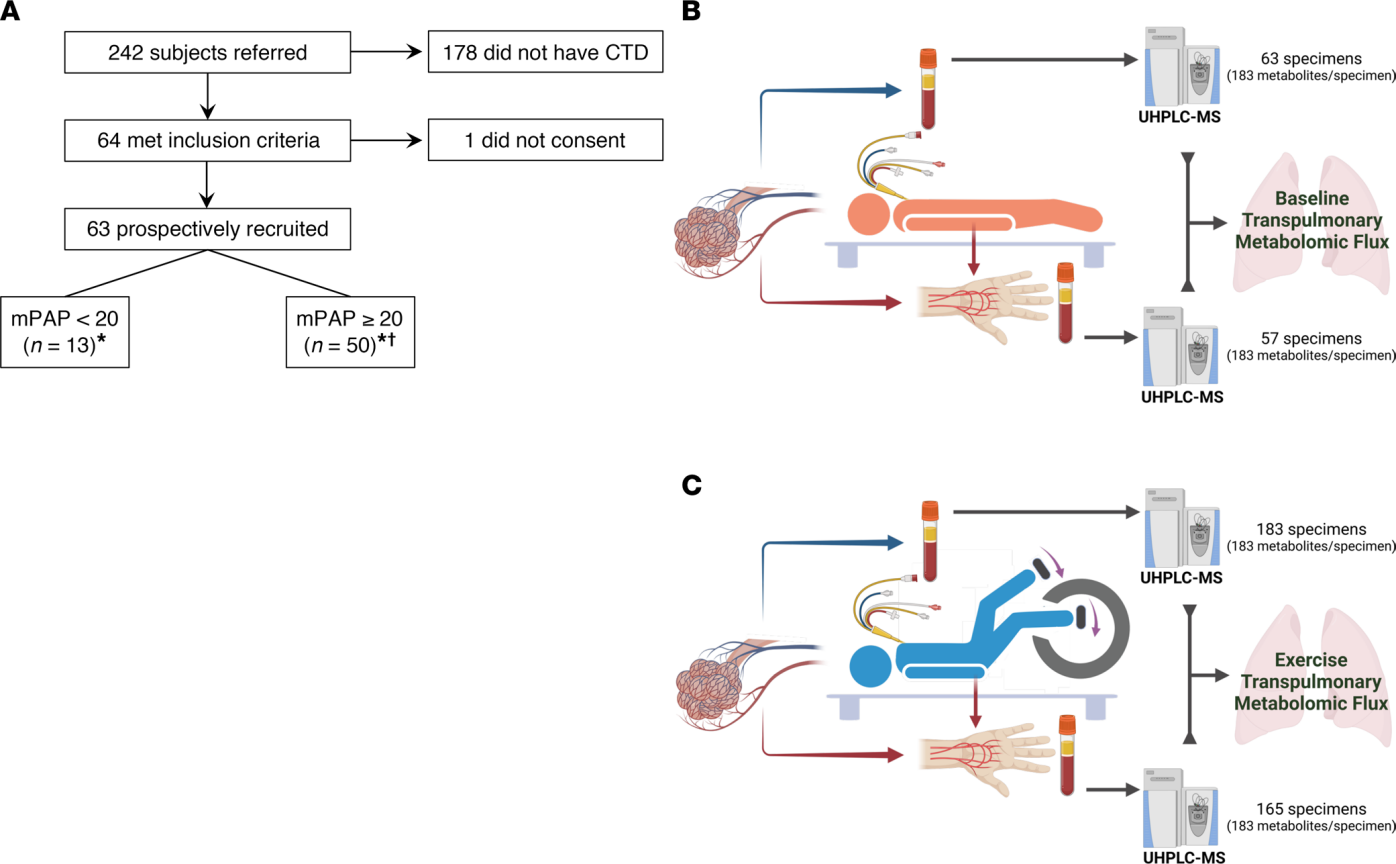
Participant enrollment and study protocol. (**A**) A flowchart illustration of prospective patient recruitment. *Three participants in the group did not have arterial access. ^†^One participant in this group was unable to exercise due to dyspnea during the RHC. (**B** and **C**) Cartoon illustrations of the study protocol at baseline (**B**) and during exercise (**C**), combining RHC, transpulmonary plasma collection, and mass spectrometry–based untargeted metabolomics. At baseline (**B**), 63 and 57 plasma specimens were collected and processed from the pulmonary and the radial arteries, respectively, accounting for 6 participants who underwent exercise without an arterial line. During exercise (**C**), 183 and 165 specimens were collected and processed from the pulmonary and the radial arteries, respectively, accounting for 1 patient who was unable to exercise, 6 participants who underwent exercise without an arterial line, and 3 participants who experienced maximal work rate during free-wheeling. All specimens were analyzed by mass spectrometry in a single batch. The cartoons were created with BioRender.com. CTD, connective tissue disease; mPAP, mean pulmonary artery pressure; UHPLC-MS, ultrahigh-pressure liquid chromatography–mass spectrometry.

We screened 242 consecutive patients referred for clinically indicated right heart catheterization (RHC) and identified 64 patients with CTD meeting the enrollment criteria, 63 of whom consented to study participation (Figure 1A and Table 1). The median age of the participants was 50 years. Most (81.0%) were female, as anticipated based on the demographic characteristics of CTD. A majority of the patients had systemic sclerosis as the etiology of their CTD, with systemic lupus erythematosus also heavily represented. Their left ventricular systolic function was preserved.

All participants underwent incremental, symptom-limited exercise RHC on a supine cycle ergometer as described in our previously published protocol (30). At baseline, 50 participants had elevated baseline mPAP > 20 mmHg, of whom 27 were on PAH-specific therapy. For the 23 untreated patients, the current study protocol represented their formal diagnostic RHC. Of the 13 participants with mPAP ≤ 20, 8 participants were not on PAH-specific therapy (i.e., absence of PAH), and the other 5 had controlled, treated PAH. None of the participants was on systemic prostacyclin-analog therapy, reflecting medication availability in São Paulo, Brazil, at the time of our study. Approximately half (47.6%) of the participants were in NYHA functional class II.

One participant was unable to complete the exercise protocol beyond the initial baseline stage because of limiting dyspnea. Three participants experienced their maximal work rate during free-wheeling; their hemodynamic and metabolite readouts during free-wheeling were treated as peak exercise variables. Six participants exercised without arterial lines due to anatomic limitations.

The participants’ exercise physiology was consistent with significant disease burden with a median work rate achieved of 30 W (Table 2 and Supplemental Table 1; supplemental material available online with this article; https://doi.org/10.1172/jci.insight.187911DS1). The increase in cardiac output during exercise was largely driven by heart rate with minimal stroke volume augmentation. These data, along with the inadequate fall in PVR, collectively reflect advanced pulmonary vascular disease state.

### Transpulmonary metabolite analysis.

**Figure 2:**
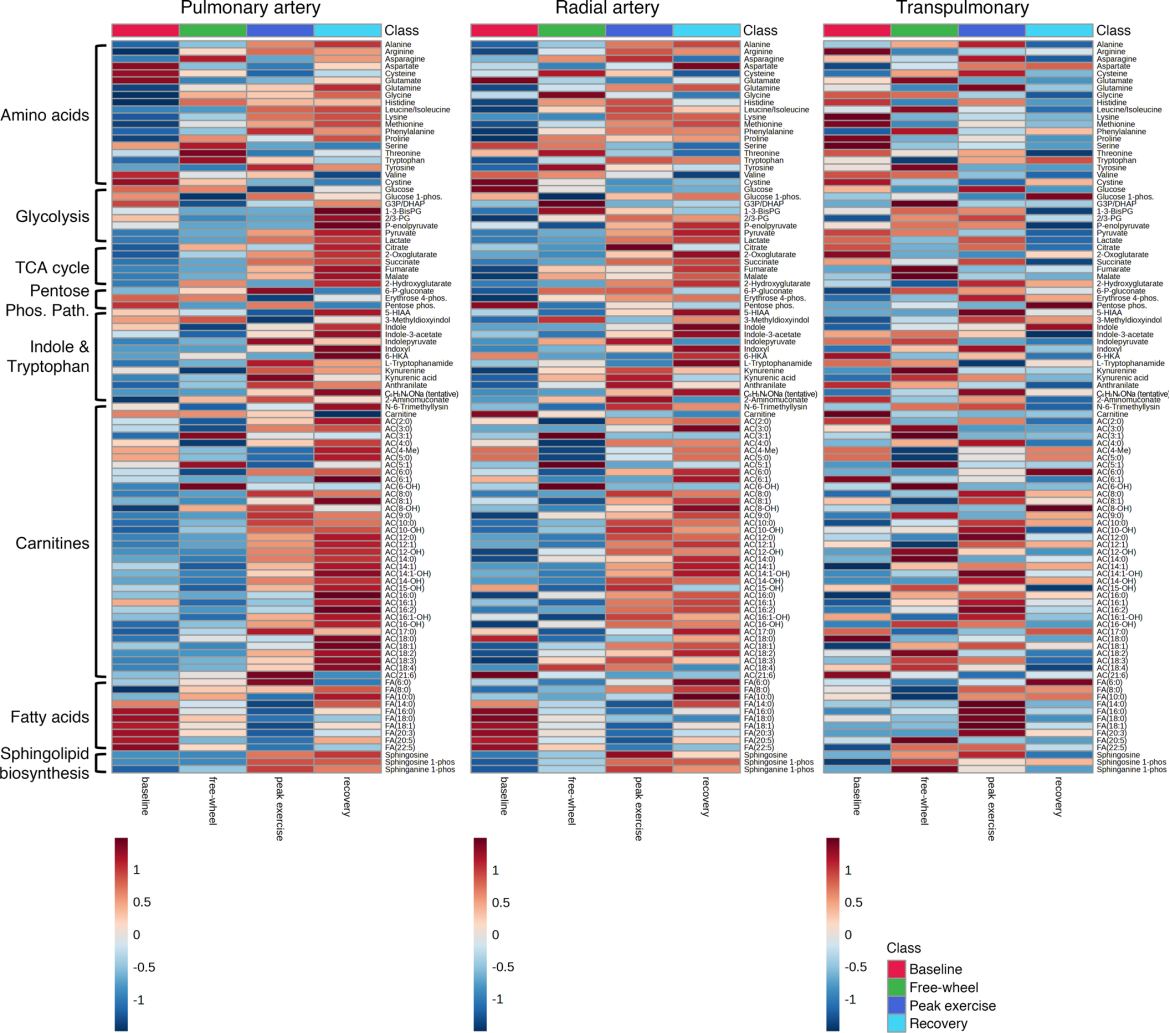
Heatmap overview of metabolite availability according to plasma collection site and exercise stage. The pulmonary artery (left; *n* = 63, 59, 62, 62 plasma samples for baseline, free-wheel, peak exercise, and recovery, respectively) and radial artery (middle; *n* = 57, 53, 56, 56 plasma samples for baseline, free-wheel, peak exercise, and recovery, respectively) heatmaps demonstrate similar patterns, including increased abundance of TCA cycle intermediates, tryptophan metabolites, and carnitines over the course of exercise (i.e., from baseline to peak exercise and recovery) and decreased availability of fatty acids during exercise. A transpulmonary analysis (right; *n* = 57, 53, 56, 56 plasma samples for baseline, free-wheel, peak exercise, and recovery, respectively), in which the pulmonary artery peak area was subtracted from the radial artery peak area for each metabolite, shows a distinct pattern of metabolite handling by the pulmonary vascular bed. The pulmonary artery analysis had 24 more samples than the other 2 analyses, representing the 6 participants who exercised without radial arterial lines. The figures were prepared using MetaboAnalyst v 6.0. Specifically, original peak areas were used and standardized by autoscaling per feature (metabolite). Each box represents the group average value. 1-3-BisPG, bisphosphoglycerate; 2/3-PG, phosphoglycerate; 5-HIAA, 5-hydroxyindoleacetate; 6-HKA, 6-hydroxykynurenic acid; 6-P-gluconate, 6-phosphogluconate; erythrose 4-phos., erythrose 4-phosphate; G3P/DHAP, glyceraldehyde 3-phosphate/dihydroxyacetone phosphate; glucose 1-phos., glucose 1-phosphate; P-enolpyruvate, phosphoenolpyruvate; pentose phos., pentose phosphates; sphinganine 1-phos., sphinganine 1-phosphate; sphingosine 1-phos., sphingosine 1-phosphate.

Ultrahigh-throughput metabolomics with a particular focus on polar metabolites in central energy and redox pathways was performed in a single batch on 468 plasma specimens: 63 participants across 4 time points and 2 collection sites (Figure 1). Accuracy of the mass spectrometric metabolite measurements was confirmed using stable isotope–labeled metabolite standards (Supplemental Table 2). In the pulmonary artery plasma, the following metabolites increased as exercise progressed: late glycolytic intermediates (phosphoglycerate, phosphoenolpyruvate, pyruvate, and lactate), TCA cycle intermediates, tryptophan catabolites, and long-chain acylcarnitines (Figure 2). Additionally, a decrease in long-chain fatty acids was observed in the pulmonary artery. We saw similar trends of metabolite abundances over the course of exercise in the radial artery plasma, suggesting that the observed patterns are reflective of whole-body metabolism, rather than specifically of pulmonary vascular biology.

Consistent with our hypothesis, transpulmonary (radial artery to pulmonary artery) analysis of metabolite levels revealed a unique pattern of pulmonary vascular metabolite handling, distinct from those observed in either artery in isolation (Figure 2). For example, at peak exercise, there was an increased net efflux of long-chain fatty acids out of the pulmonary vascular bed (i.e., more excretion and less absorption). We also observed more net efflux of long-chain acylcarnitines during free-wheeling and peak exercise and accentuated net influx of the late glycolytic intermediates into the pulmonary vasculature during recovery.

Partial least squares-discriminant analysis (PLS-DA) of transpulmonary metabolites revealed modest separations among the 4 exercise stages (Supplemental Figure 1). Among the top 25 metabolites with highest variable importance in projection (VIP) scores, indicative of greatest discriminating power among the 4 exercise stages, 9 and 5 metabolites belonged to nucleotide and fatty acid metabolisms, respectively. When the pulmonary artery (Supplemental Figure 2) and the radial artery (Supplemental Figure 3) plasma samples were studied in isolation, PLS-DA demonstrated greater separations among the 4 exercise stages. Importantly, most metabolites with high VIP scores were shared between the 2 arteries and were distinct from those generated by the transpulmonary analysis, again underscoring specificity of the transpulmonary approach to pulmonary vascular biology (Supplemental Figure 4).

**Figure 3:**
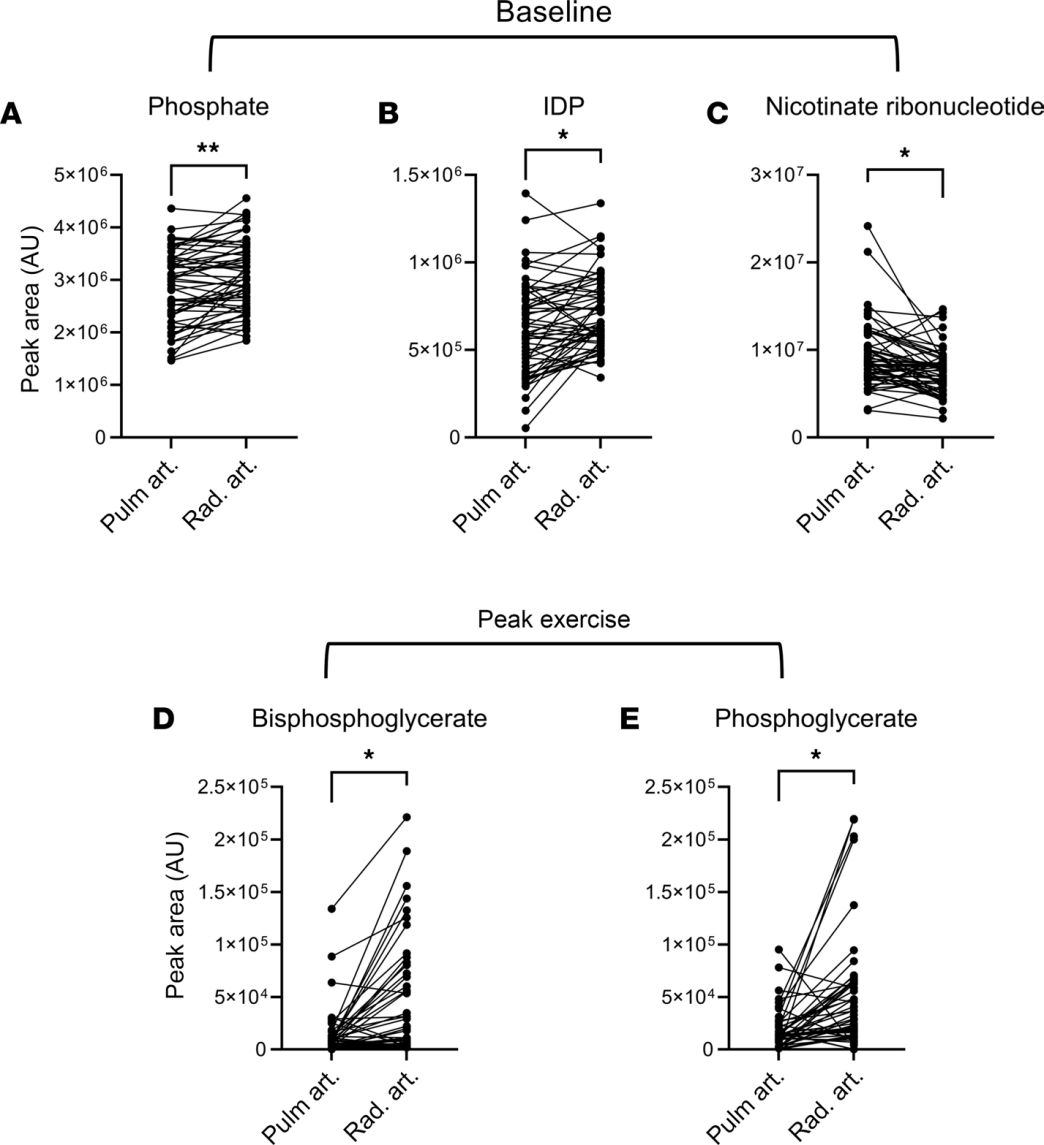
Net flux of metabolites across the pulmonary vascular bed. Net secretion and absorption of metabolites were assessed using multiple paired *t* tests, corrected for multiple comparisons and *P* values adjusted using the Holm-Šídák method. (**A**–**C**) At baseline (*n* = 57; 6 out of 63 participants without arterial lines), the pulmonary vascular bed demonstrated net flux of metabolites of the nucleotide pathway, specifically net secretion of phosphate and inosine diphosphate (IDP) and net absorption of nicotinate ribonucleotide (*P* = 0.0099, 0.0419, and 0.0434, respectively). (**D** and **E**) During peak exercise (*n* = 56), net secretion of 2 glycolysis intermediates, bisphosphoglycerate (*P* = 0.0128) and phosphoglycerate (*P* = 0.0216), was observed. **P < 0.01; *P < 0.05. AU, arbitrary unit; Pulm. Art, pulmonary artery; Rad. Art., radial artery.

We subsequently examined whether the pulmonary vascular bed demonstrated net secretion or absorption of individual metabolites (Figure 3 and Supplemental Table 3). When metabolite levels in the pulmonary artery were compared with those in the radial artery, we observed at baseline net secretion of phosphate and inosine diphosphate (IDP) and net absorption of nicotinate ribonucleotide, all 3 belonging to nucleotide metabolism. Net secretion of the 2 glycolytic intermediates — bisphosphoglycerate and phosphoglycerate — were seen during peak exercise.

### Correlations between transpulmonary metabolite gradients and hemodynamic indices.

**Figure 4:**
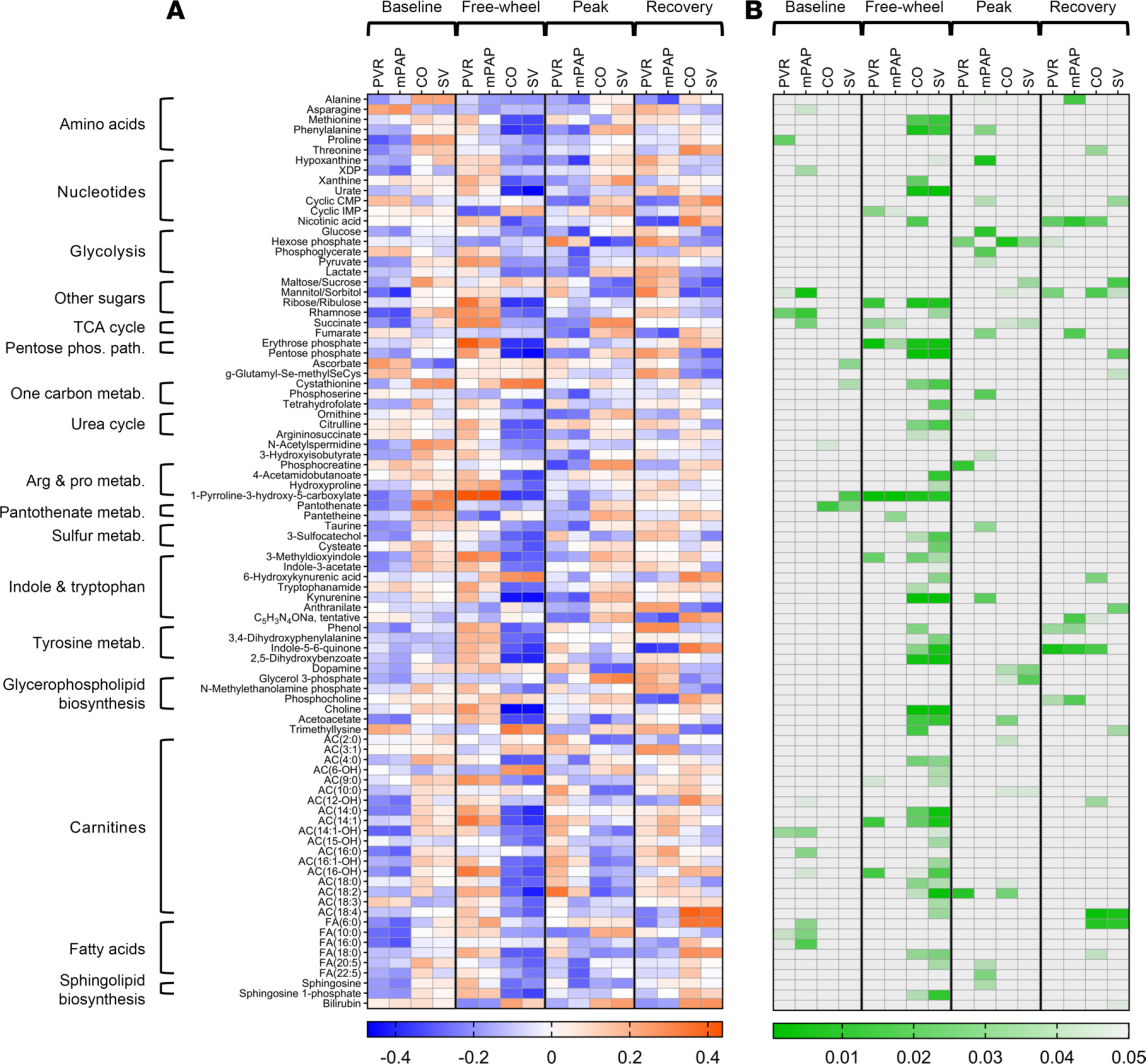
Correlations between hemodynamic indices and transpulmonary metabolite flux within each exercise stage. Spearman correlation coefficients are shown on the left (**A**), and *P* values are shown on the right (**B**). For example, peak exercise (PEAK) statistics represent correlations between hemodynamic variables and metabolites both measured during peak exercise. Spearman rank correlations were performed using MetaboAnalyst v 6.0, and only significant metabolites (*P* < 0.05 in at least 1 exercise stage for at least 1 hemodynamic endpoint) are included in the heatmaps. AC, acylcarnitine; Arg, arginine; cyclic CMP, cytidine cyclic 3′,5′ monophosphate; cyclic IMP, inosine cyclic 3′,5′ monophosphate; CO, cardiac output; FA, fatty acid; mPAP, mean pulmonary artery pressure; Pro, proline; PVR, pulmonary vascular resistance; SV, stroke volume; TCA, tricarboxylic acid; XDP, xanthosine diphosphate.

To elucidate the physiologic significance of pulmonary vascular metabolism, we first correlated transpulmonary metabolite gradients with the 4 clinically relevant hemodynamic endpoints (PVR, mPAP, stroke volume, cardiac output) within each exercise stage (Figure 4). The most significant correlations were seen during the free-wheeling stage, with the following noticeable patterns: negative correlations of stroke volume and cardiac output with tryptophan catabolites (e.g., kynurenine) and long-chain carnitines. At peak exercise, glycolytic intermediates significantly correlated with the hemodynamic variables. RVSWI, which incorporates both the right ventricular function and pulmonary vascular physiology (31), negatively correlated with transpulmonary gradients of various metabolites, largely at baseline (Supplemental Figure 5, A and B); no clear pattern was observed with maximal work rate achieved (Supplemental Figure 5, C and D).

We subsequently tested how metabolite gradients across the pulmonary vasculature at baseline may relate to exercise physiology (Supplemental Figure 6). Baseline gradients of most metabolites negatively correlated with PVR and mPAP and positively correlated with cardiac output and stroke volume. Of the 4 hemodynamic variables, mPAP had significant relationships with most metabolites. RVSWI during exercise negatively correlated with baseline transpulmonary metabolite gradients, likely reflecting a heavier representation of pulmonary vascular physiology (PVR, mPAP) in RVSWI, compared with cardiac physiology (stroke volume, cardiac output). These findings collectively suggest baseline pulmonary vascular metabolite handling has lasting physiologic implications throughout exercise.

### Subgroup analyses.

**Figure 5:**
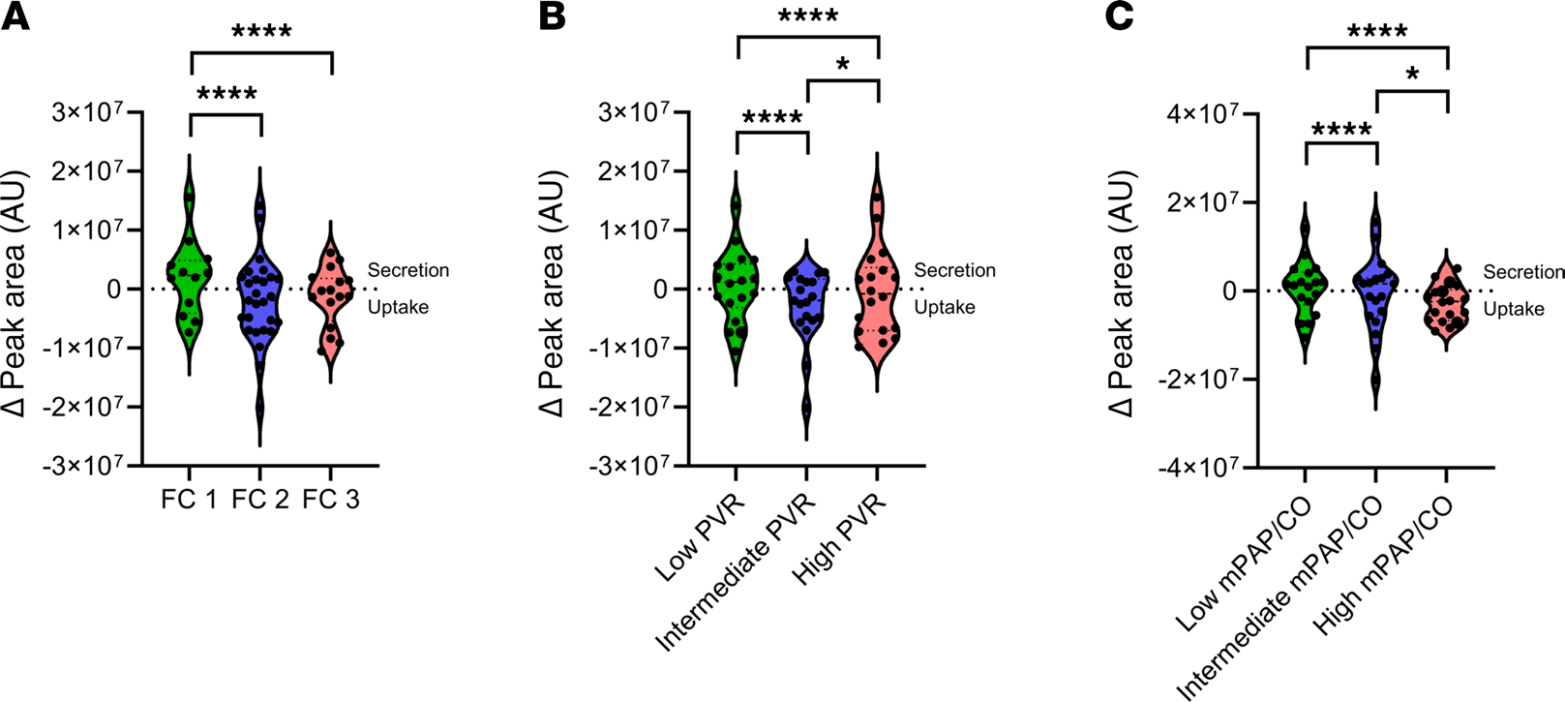
Transpulmonary lactate handling by disease severity during peak exercise. Disease severity was defined and the participants were grouped according to functional class (**A**), baseline PVR (**B**), or ΔmPAP/CO (**C**). ****P < 0.0001; *P < 0.05. Statistical analyses were performed using ordinary 2-way ANOVA with multiple comparisons corrected with the Tukey method (detailed in Supplemental Tables 3–5). In all 3 panels, a single value (6.55 × 10^–7^) significantly larger than the rest was omitted from the FC1, the low PVR, and the low mPAP/CO subgroups to aid visualization. AU, arbitrary unit; CO, cardiac output; FC, functional class; mPAP, mean pulmonary artery pressure; PVR, pulmonary vascular resistance.

The cohort was divided into subgroups based on clinically relevant metrics: baseline PVR (Supplemental Table 4 and Supplemental Figure 7), NYHA functional class (Supplemental Table 5 and Supplemental Figure 8), ΔmPAP/CO (Supplemental Table 6 and Supplemental Figure 9), and baseline mPAP (Supplemental Table 7 and Supplemental Figure 10). In these analyses, transpulmonary gradients of the following 6 metabolites consistently differentiated the subgroups, suggesting their potential pathobiologic roles in CTD-PAH: phenylalanine, inosine cyclic-3′,5′-monophosphate (cyclic IMP), lactate, FA(16:0), FA(18:0), and FA(18:1). Additionally, a few patterns were uniformly observed in these subgroup approaches. At peak exercise, there was net uptake of lactate by the pulmonary vasculature in those with more advanced disease, compared with the healthier participants with low baseline PVR, functional class 1, low ΔmPAP/CO, or normal baseline mPAP (Figure 5). Similarly, we observed more uptake of phenylalanine by the pulmonary vascular bed at peak exercise, only in those with more advanced disease as evidenced by elevated baseline PVR, functional class 3, or high ΔmPAP/CO. Analyses according to sex (Supplemental Table 8 and Supplemental Figure 11) and race or ethnic group (Supplemental Table 9 and Supplemental Figure 12) showed a similar list of metabolites differentiating the subgroups.

### Predictive analysis.

**Figure 6:**
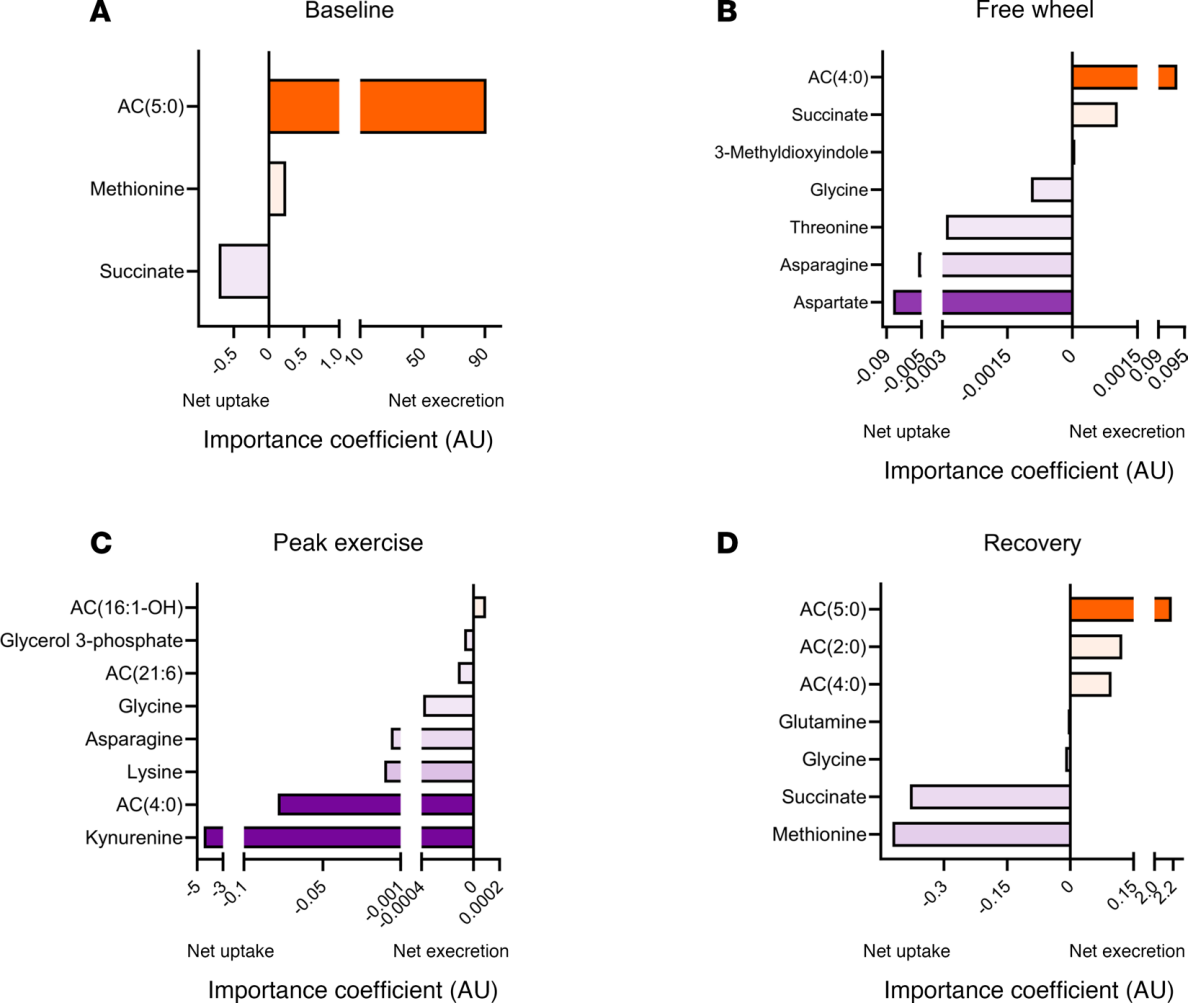
Predicting ΔmPAP/CO ratio, a surrogate for exercise-related pulmonary vascular physiologic derangement, from transpulmonary metabolite gradients at each exercise stage. (**A**–**D**) Those with negative importance coefficients (i.e., left to the abscissa of 0, depicted in purple) denote metabolites whose net uptake (or less pronounced excretion) by the pulmonary vasculature predicts mPAP/CO > 3. Conversely, those with positive importance coefficients (i.e., right to the abscissa of 0, depicted in orange) indicate metabolites whose net excretion (or less pronounced uptake) by the pulmonary vasculature predicts mPAP/CO > 3. Statistical analysis was performed using the LASSO regression model, incorporating both the metabolites measured by UHPLC-MS and the 32 redundant metabolites absolutely quantified using stable isotope–labeled standards. Demographic information (age, sex, ethnicity, smoking history, and body mass index) was also incorporated into modeling to control for possible confounding effects. AC, acylcarnitine; AU, arbitrary unit.

We investigated the ability of transpulmonary plasma metabolite gradients to predict exercise physiology, by incorporating both relatively and absolutely quantified metabolites into a least absolute shrinkage and selection operator (LASSO) regression model (Figure 6; Supplemental Table 10). Here, exercise stage–specific observations were also evident. Specifically, net excretion of short-chain acylcarnitines at baseline, free-wheeling, and recovery strongly predicted mPAP/CO > 3. On the other hand, during peak exercise, net uptake of butanoylcarnitine, AC(4:0), predicted abnormal exercise physiology, and net uptake of kynurenine was the strongest predictor of mPAP/CO > 3.

## Discussion

Detailed studies of the diseased human pulmonary vessels in PAH have been limited to deceased patients and lung transplantation recipients with advanced, severe pathology because of the high periprocedural risk in PAH (9). The pathobiology of PAH likely changes during disease progression, and therefore identifying therapeutic targets in earlier stages may prove clinically effective. Another limitation in the field is the undefined relationship between pulmonary vascular biology (e.g., metabolism) and exercise physiology. While it is increasingly recognized that pulmonary vascular hemodynamic indices during exercise are strongly prognostic in patients with CTD (32) and pulmonary vascular diseases (13–15), whether and how pathobiologically relevant metabolic pathways contribute to physical activity remain unknown. To address these limitations of traditional PAH studies, we applied untargeted, high-throughput metabolomics to transpulmonary plasma samples in adult CTD patients with suspected or established pulmonary vascular disease at 4 biologically relevant exercise stages. Utilizing this approach, we herein describe, for the first time to our knowledge, exercise stage–specific gradients of key metabolites across the pulmonary vascular bed in CTD-PAH.

The value of paired plasma collection across the pulmonary vascular bed in PAH has been previously demonstrated (17, 25, 26, 33). This transpulmonary approach enables isolation of the pulmonary vasculature without lung biopsy, thereby providing information specific to pulmonary vascular biology in earlier stages of CTD-PAH. The critical nature of such anatomic specificity is corroborated by the presence of extrapulmonary metabolic changes in PAH. In our cohort, we concordantly observed significant overlap between the pulmonary artery and the radial artery metabolite profiles (Figure 2), along with a largely shared subset of metabolites distinguishing the exercise stages in the PLS-DA (Supplemental Figure 4). These similarities suggest that metabolite profiles from either plasma collection site alone, while informative, reflect systemic metabolism, including contribution from the peripheral skeletal muscles (34).

Importantly, by quantifying transpulmonary metabolome during exercise, we demonstrate, for the first time to our knowledge, the relationship between pulmonary vascular metabolism and its physiology under stress. Although one previous metabolomics study incorporated exercise, transpulmonary metabolite analysis was strictly reported during rest (17). In the transpulmonary, correlative, subgroup, and predictive analyses of our cohort, most metabolites of significance were exercise stage specific, implying that pulmonary vascular biology and its hemodynamic effects in CTD-PAH should be considered in the context of physiologic stress. In addition to validating the prognostic implications of exercise performance in PAH (13–16), our exercise stage–specific discoveries provide insights into how pulmonary vascular metabolism may promote pulmonary hypertension and suggest a paradigm for accurately capturing the clinical impact of metabolic modulation. For instance, significant correlations between transpulmonary metabolite gradients and physiologic variables were most apparent during free-wheeling (Figure 4), which simulates day-to-day activities of mild effort. Considering that those in functional class 3 are unable to freely execute these activities, the strategy of specifically focusing on and restoring dysregulated pulmonary vascular metabolism observed during free-wheeling may effectively reduce symptom burden and improve outcomes in those with more advanced PAH.

Another consistent set of discoveries was the significance of pulmonary vascular glycolysis and lactate metabolism strictly during peak exercise. At peak exercise there was net production of lactate from the pulmonary vasculature of healthier participants with low baseline PVR, functional class 1, low ΔmPAP/CO ratio, or normal baseline mPAP (Supplemental Tables 4–7 and Figure 5), compared with net lactate uptake in those with more advanced disease and symptoms. We additionally saw net efflux of glycolytic intermediates from the pulmonary vascular bed only during peak exercise (Figure 3), and their transpulmonary gradients significantly correlated with key physiologic markers and disease severity largely at peak exercise (Figure 4).

These findings collectively suggest the need to study glycolysis inhibition in the context of maximal exercise. Glycolysis has long been considered a key pathway contributing to PAH pathobiology (6), but glycolysis-modulating therapy has not been established. Our data indicate that pulmonary vascular glycolysis is particularly elevated and analogously affects pulmonary vascular physiology during symptom-limited exercise. It is plausible that the stress of physical activity accentuates pulmonary vascular glucose metabolism, possibly secondary to inadequate oxygen availability or shear stress (35). We therefore propose assessing clinical relevance and effectiveness of glycolysis-suppressing interventions during maximal exercise, when their impact is anticipated to become most apparent.

The differential handling of lactate by the pulmonary vascular bed at peak exercise based on disease severity (Figure 5) also has important translational implications. In addition to being an end product of pyruvate fermentation (i.e., marker of glucose catabolism), lactate plays multiple biological roles (36). Notably the lungs, including lung tumors, can use lactate as an energy substrate (37, 38). Considering the accentuated influx of lactate into the pulmonary vasculature in those with more advanced diseases and the pathologic similarities between PAH and cancer (39), we hypothesize that the pulmonary vascular bed relies more heavily on lactate utilization under physiologic stress as PAH progresses. Therefore, trials examining suppression of glycolysis and lactate production may be better suited for those with less advanced PAH; the potential role of lactate in the PAH pathogenesis merits future mechanistic investigation.

Our findings synergize with other metabolomics studies of PAH. Previous plasma metabolomics analyses from a single collection site (the pulmonary artery or a peripheral vein) showed increased availability of fatty acids and acylcarnitines (18, 21, 22, 26, 27), in line with the previously demonstrated role of fatty acids in promoting pulmonary vascular remodeling (29, 40). We similarly observed separation of the 4 exercise stages by transpulmonary gradients of hexanoic acid and 4 acylcarnitines (Supplemental Figure 1). We additionally saw strong correlations between hemodynamic endpoints and the gradients of long-chain fatty acids and acylcarnitines, particularly during free-wheeling (Figure 4).

Tryptophan metabolism emerged here as a hub of dynamic pulmonary vascular metabolism. The potential contribution of the tryptophan-kynurenine pathway has been increasingly recognized in various forms of lung disease, including pulmonary hypertension (41). Tryptophan catabolites are secreted by the pulmonary vasculature in resting patients with PAH (17, 18), and their plasma levels were shown to be prognostic (42, 43). In animals, kynurenine and indoleamine 2,3-dioxygenase, the enzyme responsible for tryptophan conversion into kynurenine, attenuated pulmonary hypertension by reducing vasoconstriction (44) and vascular remodeling (45), respectively. In our cohort, kynurenine and other tryptophan catabolites significantly correlated with the hemodynamic indices throughout exercise and largely during free-wheeling (Figure 4). At peak exercise, net kynurenine uptake correlated with higher mPAP (Figure 4), and it was the strongest predictor of elevated mPAP/CO (Figure 6).

These observations collectively corroborate the clinical relevance of the tryptophan-kynurenine pathway in CTD-PAH. They additionally suggest the importance of studying the role of tryptophan catabolism in the context of physiology, particularly considering the 2 seemingly opposite ways by which tryptophan metabolism might affect the pulmonary vasculature. It is conceivable that the antiinflammatory, pro-growth effects of the tryptophan-kynurenine pathway implicated in cancer biology similarly contribute to the pulmonary vascular remodeling in CTD (46, 47). Conversely, the protective effects of kynurenine against rodent pulmonary hypertension may be relevant in the human pulmonary vasculature. While we hypothesize that the association of net kynurenine uptake with higher mPAP (Figure 4) and mPAP/CO (Figure 6) specifically during peak exercise represents accentuated kynurenine-dependent pulmonary vasodilation in the diseased state to counteract abnormally elevated mPAP during maximal exercise, understanding the precise role of the tryptophan-kynurenine pathway in CTD-PAH awaits follow-up studies.

Succinate, a TCA cycle intermediate, is another metabolite that has garnered attention for its inflammatory roles in both the cytosol and the extracellular compartment (48). In particular, succinate stabilizes hypoxia inducible factor-1α (HIF-1α), leading to accentuated inflammation. Considering that CTD-PAH is a group of inflammatory autoimmune diseases and that HIF-1α additionally contributes to pulmonary hypertension via mechanisms other than inflammation, such as glycolysis and cell survival (49–51), it is plausible that pulmonary vascular succinate handling represents a physiologically important marker of PAH. In our cohort, succinate uptake and release were key predictors of elevated mPAP/CO during exercise (Figure 6), suggesting that succinate-induced inflammation and potentially other effects of HIF-1α stabilization may significantly influence pulmonary vascular hemodynamic response to physiologic stress. The exact role of succinate in the pathobiology of CTD-PAH warrants future mechanistic studies.

Our study has several limitations. Our cohort focused on CTD and did not include healthy or CTD-free controls given ethical concerns surrounding the invasiveness of our protocol involving central venous and radial arterial punctures. Future inclusion of other comparator groups and longitudinal follow-up will clarify (a) which of the metabolomic signatures are unique to CTD or agnostic to PAH subtypes and (b) whether the observed metabolomic changes could represent beneficial, compensatory adaptations. The smaller separations of exercise stages in the transpulmonary PLS-DA (Supplemental Figure 1), which likely reflect a predominant representation of extrapulmonary (e.g., skeletal muscle) metabolism in the single-site plasma samples, may negatively impact the significance of the VIP scores. It is unclear which vascular constituent(s) (e.g., endothelium, smooth muscle cells, and so on) contribute most to the observed transpulmonary metabolomic gradients. While our transpulmonary study design is not impacted by blood draining from the coronary sinus, a small fraction of the measured transpulmonary metabolite gradients includes left myocardial metabolic activity due to the Thebesian veins, which drain the myocardium directly into the heart chambers (52). Right and left myocardial metabolisms and their relationships to cardiopulmonary exercise physiology merit dedicated investigation (25). The potential relevance of systemic manifestations of CTD during exercise, such as anaerobic oxygen utilization and alveolar hypoxia secondary to concurrent interstitial lung disease, requires further research.

The precise pathobiological and therapeutic significance of the abovementioned metabolites will require future clarification utilizing animal models and eventually clinical trials. For instance, while the shared list of metabolites differentiating the subgroups of varying PAH severity (Supplemental Tables 4–7 and Supplemental Figures 7–10) is anticipated considering that the 4 clinical parameters (baseline PVR, functional class, ΔmPAP/CO ratio, and baseline mPAP) are interrelated indices of PAH progression, whether the same metabolites have additional sex- and ethnicity-based biologic significance (Supplemental Tables 8 and 9, and Supplemental Figures 11 and 12) calls for future study. Although many of the described metabolites are typically understood in the intracellular context, most cells are known to express metabolite transporters on their cell membranes (53), providing a plausible biological rationale for their presence in the plasma, as well as for the use of extracellular circulating metabolites as a proxy for intracellular metabolic states (54). It is unlikely that the presence of these metabolites in the plasma represents spillage of intracellular contents from sheer stress-induced endothelial damage, given that many metabolites were less abundant in the radial artery compared with the pulmonary artery, an observation incompatible with pulmonary cell lysis.

In conclusion, we herein report active secretion and uptake of metabolites by the pulmonary vascular bed in patients with CTD-PAH during standardized exercise. We verify the physiologic significance of various metabolites previously shown to promote disease in animal studies and end-stage human lungs. Metabolite gradients across the pulmonary vasculature, their correlations with physiologic endpoints, subgroup analyses according to disease severity, and predictive modeling all consistently revealed exercise stage–specific differences in pulmonary vascular metabolite handling, suggesting the need to incorporate exercise into clinical studies examining metabolism modulation.

## Methods

We recently published our study protocol, including prospective patient recruitment; standardized, symptom-limited, maximal exercise RHC with radial arterial access; plasma sample collections during the 4 exercise stages (baseline, resistance-free wheeling, symptom-limited peak exercise, and recovery); and untargeted, UHPLC-MS–based metabolite extraction and quantification (Figure 1) (30).

### Sex as a biological variable.

Participants of both sexes were included in our study, and we performed a subgroup analysis based on sex.

### Patient recruitment.

Over a 24-month period, we prospectively and consecutively recruited CTD patients with suspected or confirmed diagnosis of PAH from the Pulmonary Hemodynamic Assessment Program at the UNIFESP in São Paulo, Brazil, with the following inclusion criteria: age ≥ 18 years, CTD diagnosis according to current guidelines using clinical presentation and serology, and ability to provide informed consent. Patients were excluded if any of the following criteria was met: NYHA functional class IV (symptomatic at rest), syncope or change in PAH therapy within 30 days before study, anemia (serum hemoglobin < 10 g/dL); osteoarticular limitation precluding exercise on a cycle ergometer, and pregnancy.

### Exercise RHC and transpulmonary metabolomics.

All consenting patients underwent clinically indicated, standardized, incremental, symptom-limited exercise RHC on a cycle ergometer with radial arterial lines (30), for the purpose of PAH diagnosis or risk stratification (55). All participants were required to fast for at least 4 hours before study participation. PVR was calculated from hemodynamic variables as routinely performed and previously described (31). At the 4 sequential exercise stages — baseline (rest), free-wheeling (resistance-free pedaling), peak exercise, and 2 minutes into recovery (resistance-free pedaling) — blood samples were simultaneously collected from the pulmonary artery and the systemic radial artery (i.e., across the pulmonary vascular bed). The blood samples were immediately placed on ice upon collection, and isolated plasma was stored at –80°C until analyzed.

Profiling of polar metabolites was performed using UHPLC-MS. Plasma samples were thawed on ice. Then a 10 μL aliquot was treated with 90 μL of ice-cold 5:3:2 MeOH/MeCN/water (v/v/v) with stable isotope–labeled internal standards (central carbon and nitrogen pathways from MilliporeSigma), then vortexed for 30 minutes at 4°C. Supernatants were clarified by centrifugation (10 minutes, 12,000*g*, 4°C). The resulting extracts were analyzed (5 μL/injection) on a Thermo Fisher Scientific Vanquish UHPLC coupled to a Thermo Fisher Scientific Q Exactive mass spectrometer. Metabolites were resolved using a 1-minute C18 gradient as described (56, 57) and based on earlier high-throughput methods (58). Following data acquisition, metabolites were annotated and peaks integrated using El-maven (Elucidata) in conjunction with the KEGG database and an in-house standard library, yielding 142 identifications from our library and 41 annotations. Quality control was assessed using technical replicates run at the beginning, at the end, and throughout the sequence as previously described (59).

### Statistics.

MetaboAnalyst 6.0 (https://www.metaboanalyst.ca), Prism (GraphPad Software), and Microsoft Excel were used for statistical analyses and graphing. Repeated measures statistics for hemodynamic variables from the 4 exercise stages were computed using Tukey’s multiple comparisons test on a mixed effects model. For PLS-DA, peak areas were autoscaled using MetaboAnalyst. For paired comparisons of metabolite peak areas (i.e., pulmonary artery vs. radial artery), multiple paired *t* tests in GraphPad Prism were used, corrected for multiple comparisons, and *P* values were adjusted using the Holm-Šídák method. Statistics in subgroup analyses were performed using ordinary 2-way ANOVA with multiple comparisons corrected with the Holm-Šidák (for 2 subgroups) or the Tukey (for 3 subgroups) method.

In our predictive analysis (Figure 6), we investigated the ability of transpulmonary plasma metabolite gradients at each exercise stage to predict the mPAP/CO, by taking a feature selection and dimensionality reduction approach. We first combined the metabolites measured by UHPLC-MS and the 32 redundant metabolites absolutely quantified using stable isotope–labeled standards (Supplemental Table 2). We then determined the optimal subset of these metabolites that best predicted mPAP/CO, by analyzing mPAP/CO as a binary outcome with mPAP/CO > 3 considered abnormal. Specifically, the LASSO regression model was used given its strengths in handling high-dimensional datasets and in scenarios where multicollinearity is likely. A grid search was conducted to find the optimal λ parameter for this model, and 4-fold cross-validation was used to ensure model robustness of reliability. The selection criterion was the model that maximized the area under the receiver operating characteristics curve. A separate optimal model was derived for each of the 4 exercise stages. In each model, demographic information, such as age, sex, ethnicity, smoking history, and body mass index, was incorporated into the model to control for possible confounding effects that may arise due to variations in these factors.

### Study approval.

The study was approved by the UNIFESP Institutional Ethics Committee (CEP/UNIFESP Project 0813P/2021; CAAE 49617621.1.0000.5505) and was exempt as nonhuman subject research by the UCSF Institutional Review Board. Written informed consent was received from every participant prior to study participation.

### Data availability.

Deidentified raw data are available on Metabolomics Workbench under Study ID ST003686. Individual values of presented data are separately provided in the Supporting Data Values section of the supplemental material.

## Author contributions

MHL, TCFM, RKFO, and BBG designed the study. MHL, TCFM, JAR, FIC, EVMF, JSOA, PAS, RK, CM, MMI, JLS, ACBD, DCFB, KN, RMT, PMH, AD, RKFO, and BBG (a) contributed to the acquisition, analysis, or interpretation of data for the work; (b) drafted or critically revised the work for important intellectual content; (c) approved of publication of the work; (d) and agreed to be accountable for all aspects of the work. The first 3 authors (MHL, TCFM, JAR) share the first author position, and the last 2 authors (RKFO, BBG) share the senior author position, each of whom critically and distinctively contributed to the present study. The order of the first authorship was mutually agreed upon by the first and senior authors and reflects the inception of the research hypothesis collectively by MHL and both senior authors.

## Supplementary Material

Supplemental data

Supporting data values

## Figures and Tables

**Table 1:**
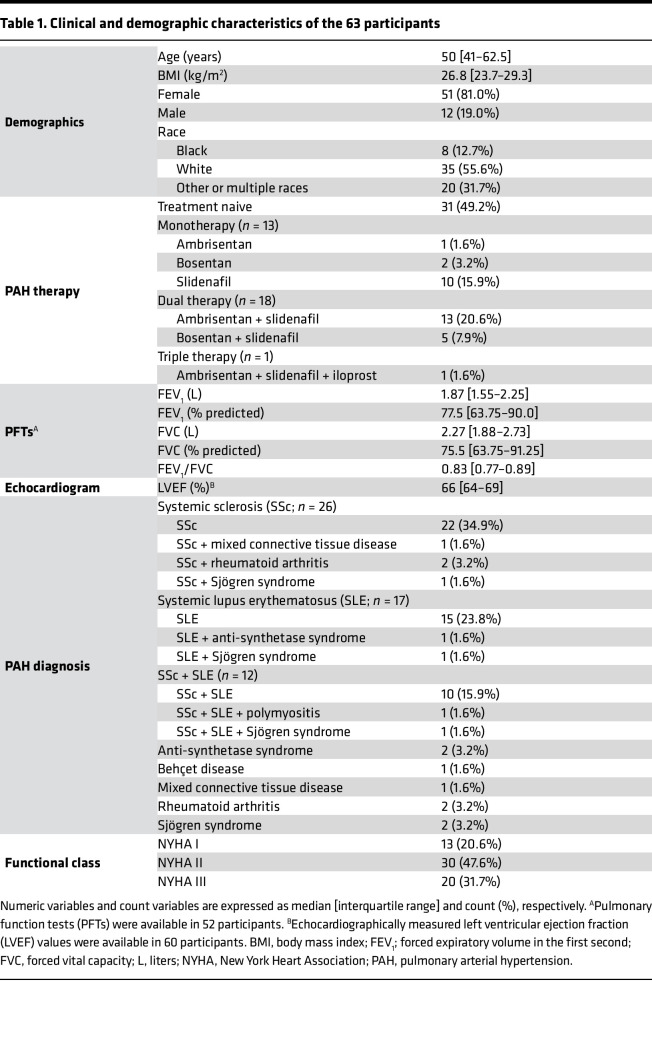
Clinical and demographic characteristics of the 63 participants

**Table 2:**
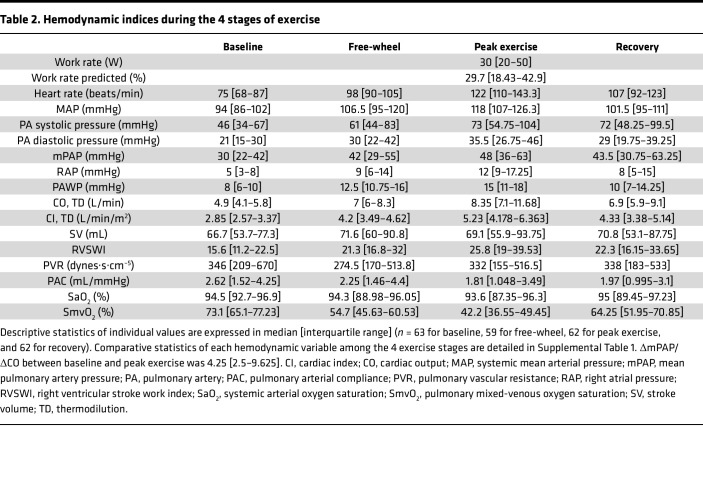
Hemodynamic indices during the 4 stages of exercise

## Version 1 05/08/2025

Electronic publication

## Version 2 05/13/2025

The authorship note was updated
