# Supplementary material for: Physiologic relevance of the transpulmonary metabolome in connective tissue disease–associated pulmonary vascular disease
Source: JCI Insight. 2025 May 8;10(9):e187911. doi: 10.1172/jci.insight.187911 (PMC12070491; doi:10.1172/jci.insight.187911)
Supplement: Supplemental data [file jciinsight-10-187911-s278.pdf]

Physiologic Relevance of Transpulmonary Metabolome in Connective Tissue Disease-Associated Pulmonary Vascular Disease

Supplemental Material

Michael H. Lee,[1,2,*] Thaís C. F. Menezes,[3,*] Julie A. Reisz,[4,*] Francesca I. Cendali,[4] Eloara V. M. Ferreira,[3] Jaquelina S. Ota-Arakaki,[3] Priscila A. Sperandio,[3] Rahul Kumar,[1,2] Claudia Mickael,[5] Martin M. Ieong,[1,2] Juliana Lucena Santos,[3] Ana Carolina B. Duarte,[3] Dara C. Fonseca Balladares,[1,2] Kevin Nolan,[1,2] Rubin M. Tuder,[5] Paul M. Hassoun,[6] Angelo D'Alessandro,[4] Rudolf K. F. Oliveira,[3,†] Brian B. Graham.[1,2,†]

1. Division of Pulmonary and Critical Care Medicine, Department of Medicine, University of California San Francisco, San Francisco, California, United States
2. Lung Biology Center, Department of Medicine, Zuckerberg San Francisco General Hospital, San Francisco, California, United States
3. Division of Respiratory Diseases, Department of Medicine, Federal University of São Paulo (UNIFESP), Hospital São Paulo, São Paulo, Brazil
4. Department of Biochemistry and Molecular Genetics, University of Colorado Anschutz Medical Campus, Aurora, Colorado, United States
5. Division of Pulmonary Sciences and Critical Care Medicine, Department of Medicine, University of Colorado Anschutz Medical Campus, Aurora, Colorado, United States
6. Division of Pulmonary and Critical Care Medicine, Department of Medicine, Johns Hopkins University School of Medicine, Baltimore, Maryland, United States

* Joint first authors
† Joint senior authors

Corresponding author:
Michael Hyunjean Lee, MD
Zuckerberg San Francisco General Hospital
University of California San Francisco
1001 Potrero Ave, Bldg 5, Room 5K1
San Francisco, CA, 94110
Email: michael.lee8@ucsf.edu

Supplemental Table 1

	Baseline	Free-Wheel	Peak Exercise	Recovery	Adjusted P Values
**Work rate (Watts)**			30 [20 - 50]		N/A
**Work rate predicted (%)**			29.7 [18.43 - 42.9]		N/A
**Heart rate (beats/min)**	75 [68 - 87]	98 [90 - 105]	122 [110 - 143.3]	107 [92 - 123]	****, ††††, ‡‡‡‡, §§§§, ####, ¶¶¶¶
**Hear rate predicted (%)**	44.3 [39.3 - 50.6]	58.3 [50.5 - 63.7]	73.2 [65.13 - 83.95]	62.45 [52.53 - 74.15]	****, ††††, ‡‡‡‡, §§§§, ####, ¶¶¶¶
**Systolic BP (mmHg)**	133 [124 - 146]	160.5 [138 - 180]	181.5 [164.8 - 200.3]	158 [142.8 - 174]	****, ††††, ‡‡‡‡, §§§§, ¶¶¶¶
**Diastolic BP (mmHg)**	73 [63 - 84]	79.5 [71 - 91]	82 [75.75 - 92.25]	73.5 [66 - 83.25]	****, ††††, ####, ¶¶¶¶
**MAP (mmHg)**	94 [86 - 102]	106.5 [95 - 120]	118 [107 - 126.3]	101.5 [95 - 111]	****, ††††, ‡‡‡, §§§§, #, ¶¶¶¶
**PA systolic pressure (mmHg)**	46 [34 - 67]	61 [44 - 83]	73 [54.75 - 104]	72 [48.25 - 99.5]	****, ††††, ‡‡‡‡, §§§§, ####, ¶¶¶¶
**PA diastolic pressure (mmHg)**	21 [15 - 30]	30 [22 - 42]	35.5 [26.75 - 46]	29 [19.75 - 39.25]	****, ††††, ‡‡‡‡, §§§§, ¶¶¶¶
**mPAP (mmHg)**	30 [22 - 42]	42 [29 - 55]	48 [36 - 63]	43.5 [30.75 - 63.25]	****, ††††, ‡‡‡‡, §§§§, ¶¶¶¶
**RAP (mmHg)**	5 [3 - 8]	9 [6 - 14]	12 [9 - 17.25]	8 [5 - 15]	****, ††††, ‡‡‡‡, §§§§, ¶¶¶¶
**PAWP (mmHg)**	8 [6 - 10]	12.5 [10.75 - 16]	15 [11 - 18]	10 [7 - 14.25]	****, ††††, ‡‡‡‡, ####, ¶¶¶¶
**CO, TD (L/min)**	4.9 [4.1 - 5.8]	7 [6 - 8.3]	8.35 [7.1 - 11.68]	6.9 [5.9 - 9.1]	****, ††††, ‡‡‡‡, §§§§, ¶¶¶¶
**CO predicted (%)**	49.4 [41.1 - 58.4]	71.7 [60.6 - 81.3]	92 [72.78 - 113.3]	78.5 [55.8 - 92.05]	****, ††††, ‡‡‡‡, §§§§, ¶¶¶¶
**CI, TD (L/min/m^2)**	2.85 [2.57 - 3.37]	4.2 [3.49 - 4.62]	5.23 [4.178 - 6.363]	4.33 [3.38 - 5.14]	****, ††††, ‡‡‡‡, §§§§, ¶¶¶¶
**SV (mL)**	66.7 [53.7 - 77.3]	71.6 [60 - 90.8]	69.1 [55.9 - 93.75]	70.8 [53.1 - 87.75]	****, †††, ‡‡
**SVi (mL/m^2)**	38.38 [32.53 - 43.56]	42.84 [36.1- 50.66]	41.17 [35.13 - 51.71]	41.14 [32.7 - 50.77]	****, †††, ‡‡
**RVSWI**	15.6 [11.2 - 22.5]	21.3 [16.8 - 32]	25.8 [19 - 39.53]	22.3 [16.15 - 33.65]	****, ††††, ‡‡‡‡, §§§, ¶¶
**PVR (dynes·s·cm^{-5})**	346 [209 - 670]	274.5 [170 - 513.8]	332 [155 - 516.5]	338 [183 - 533]	**, †††, ##, ¶¶¶
**PVRi (dynes·s·cm^{-5}·m^2)**	598 [380 - 1107]	472 [278.3 - 849.3]	546 [284.3 - 805.8]	581 [334 - 882]	**, †††, #, ¶¶¶
**PAC (mL/mmHg)**	2.62 [1.52 - 4.25]	2.25 [1.46 - 4.4]	1.81 [1.048 - 3.49]	1.97 [0.995 - 3.1]	†, ‡‡, §, #
**SVR (dynes·s·cm^{-5})**	1400 [1191 - 1749]	1116 [866.5 - 1363]	971 [683.5 - 1204]	1043 [804 - 1318]	****, ††††, ‡‡‡‡, §§§§, #, ¶
**SVRi (dynes·s·cm^{-5}·m^2)**	2489 [1942 - 2904]	1847 [1550 - 2205]	1611 [1162 - 1920]	1795 [1344 - 2273]	****, ††††, ‡‡‡‡, §§§§, ¶¶
**SaO_2 (%)**	94.5 [92.7 - 96.9]	94.3 [88.98 - 96.05]	93.6 [87.35 - 96.3]	95 [89.45 - 97.23]	**, †, #, ¶¶
**$SmvO_2$ (%)**	73.1 [65.1 - 77.23]	54.7 [45.63 - 60.53]	42.2 [36.55 - 49.45]	64.25 [51.95 - 70.85]	****, ††††, ‡‡‡‡, §§§§, ####, ¶¶¶¶
**ΔmPAP/ΔCO**		4.25 [2.5 - 9.625]			N/A

Supplemental Table 2

compound	CmpdID	Pathway	R^2	p value	Significant?
Alanine	C00041	Amino acids	0.7957	<0.0001	****
Arginine	C00062	Amino acids	0.8361	<0.0001	****
Asparagine	C00152	Amino acids	0.4094	<0.0001	****
Aspartate	C00049	Amino acids	0.391	<0.0001	****
Glutamate	C00025	Amino acids	0.8242	<0.0001	****
Glutamine	C00064	Amino acids	0.5563	<0.0001	****
Glycine	C00037	Amino acids	0.2203	<0.0001	****
Histidine	C00135	Amino acids	0.615	<0.0001	****
Leucine/Isoleucine	C00123	Amino acids	0.6548	<0.0001	****
Lysine	C00047	Amino acids	0.6445	<0.0001	****
Methionine	C00073	Amino acids	0.1122	<0.0001	****
Phenylalanine	C00079	Amino acids	0.7682	<0.0001	****
Proline	C00148	Amino acids	0.9114	<0.0001	****
Serine	C00065	Amino acids	0.2856	<0.0001	****
Threonine	C00188	Amino acids	0.7508	<0.0001	****
Tryptophan	C00078	Amino acids	0.1477	<0.0001	****
Tyrosine	C00082	Amino acids	0.2374	<0.0001	****
Valine	C00183	Amino acids	0.7798	<0.0001	****
Pyruvate	C00022	Glycolysis	0.8061	<0.0001	****
Lactate	C01432	Glycolysis	0.8216	<0.0001	****
Citrate	C00158	TCA cycle	0.664	<0.0001	****
Succinate	C00042	TCA cycle	0.7692	<0.0001	****
Kynurenine	C00328	Indole and Tryptophan	0.8538	<0.0001	****
Carnitine	C00318	Carnitine and fatty acid metabolism	0.6929	<0.0001	****
AC(2:0)	HMDB0000201	Carnitine and fatty acid metabolism	0.8746	<0.0001	****
AC(3:0)	HMDB0000824	Carnitine and fatty acid metabolism	0.9295	<0.0001	****
AC(4:0)	HMDB0002013	Carnitine and fatty acid metabolism	0.07256	<0.0001	****
AC(5:0)	HMDB0000688	Carnitine and fatty acid metabolism	0.9337	<0.0001	****
AC(8:0)	HMDB0000791	Carnitine and fatty acid metabolism	0.9565	<0.0001	****
AC(14:0)	HMDB0005066	Carnitine and fatty acid metabolism	0.9382	<0.0001	****
AC(16:0)	HMDB0000222	Carnitine and fatty acid metabolism	0.8501	<0.0001	****
FA(16:0)	C00249	Saturated Fatty acids	0.748	<0.0001	****

Supplemental Table 3

	Baseline			Peak Exercise	
**Metabolite**	**Phosphate**	**IDP**	**Nicotinate ribonucleotide**	**Bisphosphoglycerate**	**Phosphoglycerate**
**Mean (PA)**	2837736	617244	9270755	12286	16481
**SD (PA)**	707589	268196	3750873	22187	18821
**Mean (Rad)**	3076357	717479	7452944	37966	45977
**SD (Rad)**	667176	211917	2633560	52945	53596
**SD (Pooled)**	695039	245837	3353032	42417	42642
**Effect size**	0.343320303	0.407729512	0.542139473	0.605417639	0.691712396

Supplemental Table 4

		**Low Baseline PVR**<br>**166 [109 - 240] dynes·s·cm^{-5}**	**Intermediate Baseline PVR**<br>**357 [313 - 428] dynes·s·cm^{-5}**	**High Baseline PVR**<br>**774 [690 - 1022] dynes·s·cm^{-5}**	**Adjusted P Value**
**Phenylalanine**	Baseline	8.70E+05 [-2.05E+06 – 2.39E+06]	4.64E+05 [-1.47E+06 – 2.08E+06]	1.15E+06 [-2.62E+06 –1.96E+06]	ns
	Free-Wheel	3.32E+05 [-1.79E+06 – 1.96E+06]	1.74E+06 [-2.78E+06 – 5.17E+06]	1.74E+06 [-4.34E+06 – 4.08E+06]	ns
	Peak Exercise	1.03E+06 [-1.10E+06 – 3.01E+06]	3.63E+05 [-2.19E+06 – 1.93E+06]	-9.88E+05 [-3.43E+06 –1.34E+06]	††
	Recovery	1.01E+06 [-2.20E+06 – 3.91E+06]	6.95E+05 [-8.71E+05 – 3.46E+06]	-5.99E+05 [-4.48E+06 – 3.16E+06]	ns
**Cyclic IMP**	Baseline	1.92E+06 [-3.40E+06 – 1.52E+07]	-7.33E+05 [-3.74E+06 – 6.80E+06]	1.60E+06 [-9.30E+05 – 1.24E+07]	****, §§§§
	Free-Wheel	2.99E+06 [-9.22E+05 – 6.52E+06]	1.32E+06 [-4.10E+06 – 6.87E+06]	-2.05E+06 [-9.38E+06 – 7.25E+06]	††
	Peak Exercise	6.26E+04 [-6.06E+06 – 5.39E+06]	1.85E+06 [-3.01E+06 – 6.88E+06]	7.57E+05 [-5.11E+06 – 4.87E+06]	ns
	Recovery	-6.25E+05 [-6.82E+06 – 1.89E+06]	-1.07E+06 [-4.45E+06 – 3.33E+06]	-1.01E+06 [-4.95E+06 – 4.98E+06]	§§
**Lactate**	Baseline	3.78E+05 [-2.89E+05 – 1.82E+06]	-2.95E+05 [-9.53E+05 – 2.22E+05]	3.43E+05 [-9.30E+05 – 1.65E+06]	ns
	Free-Wheel	-4.70E+05 [-2.78E+06 – 8.92E+05]	-8.68E+05 [-2.38E+06 – 1.64E+06]	5.43E+04 [-1.85E+06 – 1.66E+06]	ns
	Peak Exercise	1.49E+06 [-2.34E+06 – 4.95E+06]	-1.91E+06 [-5.29E+06 – 1.70E+06]	-7.78E+05 [-7.02E+06 – 3.68E+06]	****, ††††, §
	Recovery	-2.71E+06 [-4.97E+06 – 1.24E+06]	-1.47E+06 [-6.88E+06 – 6.22E+06]	5.60E+05 [-4.41E+06 – 5.59E+06]	*
**FA(16:0)**	Baseline	8.46E+06 [1.81E+06 – 1.64E+07]	-1.54E+06 [-1.27E+07 – 6.21E+06]	-6.87E+06 [-2.92E+07 – 2.66E+06]	**, ††††, §§§§
	Free-Wheel	6.54E+05 [-1.19E+07 – 3.45E+06]	5.28E+05 [-1.10E+07 – 1.26E+07]	-2.53E+06 [-2.26E+07 – 1.39E+07]	****, §§§§
	Peak Exercise	-4.73E+05 [-6.93E+06 – 1.87E+07]	2.32E+06 [-4.85E+06 – 1.04E+07]	3.24E+06 [-1.17E+06 – 1.24E+07]	ns
	Recovery	1.27E+06 [-9.38E+06 – 1.39E+07]	6.34E+06 [-8.45E+06 – 9.50E+06]	-5.36E+06 [-1.71E+07 – 2.53E+06]	††††, §§§§
**FA(18:0)**	Baseline	2.46E+06 [-3.41E+06 – 7.34E+06]	1.92E+05 [-5.71E+06 – 3.47E+06]	5.96E+05 [-2.99E+06 – 3.60E+06]	ns
	Free-Wheel	-1.64E+06 [-6.70E+06 – 5.38E+06]	-8.57E+05 [-3.85E+06 – 4.65E+06]	1.53E+06 [-3.29E+06 – 5.85E+06]	ns
	Peak Exercise	1.59E+06 [-3.05E+06 – 1.03E+07]	2.53E+06 [-8.89E+05 – 5.54E+06]	3.43E+06 [-4.28E+06 – 5.96E+06]	†
	Recovery	1.28E+06 [-3.54E+06 – 5.01E+06]	-2.46E+05 [-2.04E+06 – 3.28E+06]	-2.80E+06 [-5.03E+06 – 2.54E+06]	††, §§
**FA(18:1)**	Baseline	1.23E+07 [-3.53E+07 – 2.55E+07]	-1.08E+06 [-7.74E+06 – 7.34E+06]	-4.68E+06 [-2.19E+07 – 2.22E+07]	††††, §§§§
	Free-Wheel	-7.33E+06 [-4.07E+07 – 1.01E+07]	8.59E+05 [-9.89E+06 – 2.39E+07]	-2.23E+06 [-2.41E+07 – 1.86E+07]	****, ††††, §§§§
	Peak Exercise	2.27E+06 [-8.94E+06 – 2.82E+07]	1.83E+06 [-6.30E+06 – 7.58E+06]	1.25E+06 [-6.09E+06 – 2.29E+07]	****, §§§§
	Recovery	7.35E+06 [-2.21E+07 – 1.96E+07]	1.12E+06 [-5.44E+06 – 1.49E+07]	7.33E+05 [-1.88E+07 – 2.80E+07]	**, ††

Supplemental Table 5

		FC 1	FC 2	FC 3	Adjusted P Value
**Phenylalanine**	Baseline	-1.39E+05 [-3.79E+06 – 2.01E+06]	1.27E+06 [-2.97E+05 – 2.13E+06]	4.60E+05 [-3.21E+06 – 1.65E+06]	ns
	Free-Wheel	1.69E+05 [-1.42E+06 – 2.89E+06]	1.51E+06 [-2.52E+06 – 2.88E+06]	1.29E+06 [-3.08E+06 – 4.87E+06]	ns
	Peak Exercise	1.03E+06 [-1.22E+06 – 5.26E+06]	4.81E+05 [-2.19E+06 – 1.79E+06]	-1.89E+06 [-4.12E+06 – 1.67E+06]	**, ††††, §§§§
	Recovery	1.01E+06 [-7.56E+05 – 3.17E+06]	6.15E+05 [-1.04E+06 – 4.23E+06]	-8.92E+05 [-3.65E+06 – 2.67E+06]	ns
**Cyclic IMP**	Baseline	1.92E+06 [-4.79E+06 – 1.27E+07]	-7.11E+05 [-2.96E+06 – 7.32E+06]	3.28E+04 [-1.67E+06 – 1.22E+07]	**, §§§§
	Free-Wheel	3.32E+06 [-1.96E+06 –6.22E+06]	1.13E+06 [-4.88E+06 –7.24E+06]	-1.90E+06 [-8.65E+06 – 7.07E+06]	†
	Peak Exercise	1.70E+06 [-7.24E+06 – 5.79E+06]	9.01E+05 [-3.25E+06 – 8.28E+06]	4.57E+05 [-5.54E+06 – 4.37E+06]	*, §§
	Recovery	1.39E+06 [-2.14E+06 – 6.40E+06]	-3.64E+05 [-7.51E+06 – 3.92E+06]	-4.10E+06 [-6.43E+06 – -1.55E+06]	****, ††††
**Lactate**	Baseline	-2.24E+05 [-4.94E+05 – 5.61E+05]	9.06E+04 [-9.96E+05 – 5.07E+05]	3.78E+05 [-7.09E+05 – 1.67E+06]	ns
	Free-Wheel	6.48E+05 [-2.04E+06 – 1.73E+06]	-6.34E+05 [-2.07E+06 – 2.52E+06]	-6.65E+05 [-4.28E+06 – 5.36E+05]	ns
	Peak Exercise	2.71E+06 [-3.47E+06 – 6.62E+06]	-1.91E+06 [-6.97E+06 – 2.01E+06]	-7.65E+05 [-5.49E+06 – 1.84E+06]	****, ††††
	Recovery	-2.71E+06 [-5.28E+06 – 3.81E+06]	-1.52E+06 [-4.97E+06 – 3.55E+06]	-1.29E+06 [-5.93E+06 – 8.36E+06]	ns
**FA(16:0)**	Baseline	8.01E+06 [5.26E+05 – 1.12E+07]	-4.18E+06 [-2.91E+07 – 1.20E+07]	-1.50E+06 [-1.24E+07 – 3.62E+06]	****, ††††
	Free-Wheel	1.09E+06 [-8.93E+06 – 8.34E+06]	-6.21E+05 [-1.32E+07 – 1.34E+07]	-3.58E+06 [-1.55E+07 – 7.22E+06]	††, §§§§
	Peak Exercise	5.55E+06 [-5.92E+06 – 2.15E+07]	1.46E+06 [-3.26E+06 – 1.23E+07]	-4.62E+05 [-1.04E+07 – 9.08E+06]	****, ††††
	Recovery	-2.33E+06 [-1.72E+07 – 1.13E+07]	-4.81E+06 [-1.24E+07 – 9.44E+06]	2.03E+06 [-1.42E+07 – 1.55E+07]	ns
**FA(18:0)**	Baseline	5.96E+05 [-3.80E+06 – 7.11E+06]	1.11E+06 [-7.73E+06 – 4.76E+06]	8.63E+05 [-2.83E+06 – 4.03E+06]	ns
	Free-Wheel	-1.64E+06 [-6.49E+06 – 4.26E+06]	3.67E+05 [-3.03E+06 – 5.07E+06]	9.37E+05 [-6.60E+06 – 5.58E+06]	ns
	Peak Exercise	3.27E+06 [7.53E+04 – 1.10E+07]	3.23E+06 [-3.09E+06 – 6.80E+06]	4.22E+05 [-4.30E+06 – 5.80E+06]	*, †††
	Recovery	-2.96E+05 [-5.47E+06 – 4.45E+06]	6.49E+05 [-2.51E+06 – 4.47E+06]	-5.75E+05 [-4.09E+06 – 1.82E+06]	ns
**FA(18:1)**	Baseline	9.02E+06 [-1.27E+07 – 4.08E+07]	3.62E+06 [-1.32E+07 – 1.87E+07]	-2.40E+06 [-2.08E+07 – 1.77E+07]	****, ††††, §§
	Free-Wheel	-3.13E+06 [-1.07E+07 – 6.47E+06]	-4.30E+06 [-2.29E+07 – 1.80E+07]	1.05E+06 [-3.02E+07 – 2.00E+07]	**, ††††, §§§§
	Peak Exercise	-3.34E+05 [-9.92E+06 – 3.40E+07]	-4.34E+05 [-6.63E+06 – 8.70E+06]	5.58E+06 [-2.06E+06 – 3.30E+07]	****, ††, §§§§
	Recovery	5.55E+06 [-3.15E+07 – 1.87E+07]	6.11E+06 [-5.19E+06 – 1.80E+07]	-3.59E+06 [-1.94E+07 – 1.28E+07]	****, §§§§

Supplemental Table 6

		Low mPAP/CO 2.3 [1.6 - 2.6]	Intermediate mPAP/CO 4.4 [3.9 - 6.0]	High mPAP/CO 12.0 [9.9 - 17.3]	Adjusted P Value
**Phenylalanine**	Baseline	1.21E+06 [-2.05E+06 – 2.39E+06]	6.41E+05 [-3.12E+06 – 2.08E+06]	7.98E+05 [-9.81E+05 – 1.75E+06]	ns
	Free-Wheel	-1.77E+05 [-1.79E+06 – 1.96E+06]	1.35E+06 [-3.93E+06 – 3.22E+06]	2.48E+06 [-1.02E+06 – 4.96E+06]	ns
	Peak Exercise	7.71E+05 [-2.00E+06 – 2.25E+06]	1.21E+06 [-4.19E+05 – 6.26E+06]	-1.39E+06 [-3.54E+06 – 6.15E+05]	††, §§
	Recovery	1.01E+06 [-1.64E+06 – 3.91E+06]	-8.71E+05 [-2.12E+06 – 1.92E+06]	5.19E+05 [-1.88E+06 – 4.33E+06]	ns
**Cyclic IMP**	Baseline	6.80E+06 [-3.40E+06 – 1.52E+07]	-1.18E+05 [-3.74E+06 – 7.90E+06]	-7.52E+05 [-1.96E+06 – 1.24E+06]	**, ††††
	Free-Wheel	2.99E+06 [-1.75E+06 – 8.05E+06]	-9.22E+05 [-8.25E+06 – 4.55E+06]	2.33E+06 [-6.83E+06 – 7.29E+06]	****
	Peak Exercise	2.66E+06 [-4.01E+06 – 5.46E+06]	-1.06E+06 [-6.88E+06 – 5.68E+06]	1.34E+06 [-3.66E+06 – 5.17E+06]	ns
	Recovery	-4.95E+05 [-6.13E+06 – 1.89E+06]	-1.53E+06 [-4.45E+06 – 6.23E+06]	-1.81E+06 [-5.14E+06 – 2.24E+06]	††††, §§§§
**Lactate**	Baseline	3.40E+05 [-2.89E+05 – 1.82E+06]	-1.96E+05 [-8.40E+05 – 3.70E+05]	-1.61E+04 [-9.97E+05 – 1.27E+06]	ns
	Free-Wheel	-5.08E+05 [-2.78E+06 – 6.48E+05]	1.17E+06 [-1.55E+06 – 3.44E+06]	-1.11E+06 [-2.54E+06 – 4.96E+05]	ns
	Peak Exercise	1.49E+06 [-2.34E+06 – 4.95E+06]	1.62E+06 [-5.63E+06 – 2.97E+06]	-2.38E+06 [-6.68E+06 – 2.08E+05]	****, ††††, §
	Recovery	-2.95E+06 [-6.19E+06 – 2.98E+06]	1.62E+06 [-4.79E+06 – 5.29E+06]	-1.49E+06 [-4.41E+06 – 8.82E+06]	*, †
**FA(16:0)**	Baseline	7.82E+06 [-3.72E+06 – 1.24E+07]	-4.32E+06 [-2.03E+07 – 8.01E+06]	-3.49E+06 [-1.65E+07 – 3.54E+06]	****, ††††
	Free-Wheel	6.54E+05 [-2.22E+07 – 3.45E+06]	8.48E+05 [-1.04E+07 – 1.80E+07]	-4.62E+06 [-2.53E+07 – 4.21E+06]	****, ††, §§§§
	Peak Exercise	-4.73E+05 [-1.51E+07 – 1.47E+07]	6.16E+06 [-4.48E+06 – 1.78E+07]	8.15E+04 [-3.95E+06 – 8.76E+06]	****, ††, §§§§
	Recovery	5.62E+06 [-1.64E+07 – 1.39E+07]	-3.07E+06 [-1.24E+07 – 9.44E+06]	-4.90E+06 [-1.34E+07 – 6.08E+06]	*, ††††, §§§§
**FA(18:0)**	Baseline	1.81E+06 [-4.19E+06 – 8.47E+06]	1.11E+06 [-5.71E+06 – 4.04E+06]	8.56E+05 [-3.38E+06 – 2.64E+06]	ns
	Free-Wheel	-8.72E+05 [-3.92E+06 – 3.38E+06]	1.12E+06 [-3.78E+06 – 5.14E+06]	1.53E+06 [-6.26E+06 – 5.54E+06]	*
	Peak Exercise	1.59E+06 [-4.72E+06 – 6.80E+06]	3.37E+06 [-8.89E+05 – 8.30E+06]	3.37E+06 [-2.01E+06 – 5.63E+06]	****, §§
	Recovery	1.93E+06 [-4.76E+06 – 5.04E+06]	-5.78E+05 [-2.51E+06 – 2.76E+06]	-2.63E+06 [-4.72E+06 – 1.44E+06]	†
**FA(18:1)**	Baseline	5.77E+06 [-3.53E+07 – 1.89E+07]	4.36E+06 [-1.31E+07 – 2.02E+07]	-1.74E+06 [-1.54E+07 – 1.15E+07]	****, ††, §§§§
	Free-Wheel	5.13E+05 [-4.07E+07 – 1.15E+07]	-5.31E+06 [-2.22E+07 – 9.36E+06]	4.88E+06 [-1.42E+07 – 1.99E+07]	****, ††††
	Peak Exercise	8.18E+05 [-6.63E+06 – 8.89E+06]	6.46E+06 [-8.56E+06 – 3.99E+07]	1.75E+06 [-4.68E+06 – 8.98E+06]	****, §§§§
	Recovery	7.35E+06 [-2.40E+07 – 1.96E+07]	5.55E+06 [-3.31E+06 – 1.77E+07]	-5.31E+06 [-2.38E+07 – 1.31E+07]	****, †††, §§§§

Supplemental Table 7

		mPAP ≤ 20<br>16.00 [14.75 - 18.00]	mPAP > 20<br>36.00 [26.00 - 46.00]	Adjusted P Value
**Cyclic IMP**	Baseline	7.77E+06 [-7.02E+06 – 1.61E+07]	-4.49E+05 [-2.00E+06 – 7.32E+06]	ns
	Free-Wheel	5.58E+06 [-8.24E+05 – 9.19E+06]	1.01E+06 [-4.32E+06 – 6.37E+06]	**
	Peak Exercise	3.43E+06 [-6.00E+06 – 5.71E+06]	7.57E+05 [-5.11E+06 – 5.17E+06]	ns
	Recovery	1.44E+06 [-1.41E+06 – 5.55E+06]	-1.41E+06 [-6.11E+06 – 2.16E+06]	*
**Lactate**	Baseline	9.14E+05 [-1.82E+05 – 2.54E+06]	-9.21E+03 [-9.30E+05 – 5.07E+05]	ns
	Free-Wheel	-5.75E+03 [-2.65E+06 – 1.01E+06]	-3.76E+05 [-2.27E+06 – 1.17E+06]	ns
	Peak Exercise	3.03E+06 [1.43E+05 – 9.62E+06]	-1.25E+06 [-5.87E+06 – 1.99E+06]	****
	Recovery	-2.83E+06 [-5.84E+06 – 3.07E+06]	-1.29E+06 [-5.02E+06 – 5.41E+06]	ns
**FA(16:0)**	Baseline	9.43E+06 [7.01E+06 – 1.64E+07]	-2.80E+06 [-2.03E+07 – 6.21E+06]	****
	Free-Wheel	-3.34E+06 [-3.13E+07 – 1.12E+06]	-6.29E+05 [-1.15E+07 – 1.20E+07]	****
	Peak Exercise	6.62E+06 [-5.05E+06 – 1.97E+07]	1.98E+06 [-5.35E+06 – 1.10E+07]	*
	Recovery	3.44E+06 [-1.11E+07 – 2.20E+07]	-2.37E+06 [-1.34E+07 – 9.45E+06]	****
**FA(18:0)**	Baseline	6.02E+06 [-1.61E+06 – 1.06E+07]	8.49E+05 [-4.57E+06 – 3.60E+06]	ns
	Free-Wheel	-3.03E+06 [-6.94E+06 – -5.68E+05]	1.12E+06 [-3.78E+06 – 5.38E+06]	*
	Peak Exercise	2.29E+06 [-4.05E+05 – 8.73E+06]	3.16E+06 [-2.27E+06 – 5.96E+06]	ns
	Recovery	1.02E+06 [-6.26E+06 – 3.98E+06]	-2.71E+05 [-3.59E+06 – 4.04E+06]	ns
**FA(18:1)**	Baseline	1.25E+07 [-2.28E+07 – 3.11E+07]	9.88E+04 [-1.38E+07 – 1.70E+07]	****
	Free-Wheel	-1.31E+06 [-4.25E+07 – 1.04E+07]	-2.23E+06 [-1.64E+07 – 1.97E+07]	****
	Peak Exercise	4.39E+06 [-9.43E+06 – 1.74E+07]	1.25E+06 [-6.01E+06 – 1.27E+07]	**
	Recovery	6.73E+06 [-1.43E+07 – 2.71E+07]	1.50E+06 [-1.58E+07 – 1.63E+07]	ns

Supplemental Table 8

		Female	Male	Adjusted P Value
**Phenylalanine**	Baseline	5.50E+05 [-2.19E+06 – 2.10E+06]	1.21E+06 [4.64E+05 – 1.96E+06]	ns
	Free-Wheel	3.32E+05 [-2.78E+06 – 2.46E+06]	4.17E+06 [1.43E+06 – 5.90E+06]	ns
	Peak Exercise	-1.14E+05 [-2.63E+06 – 2.11E+06]	1.01E+06 [-1.46E+06 – 5.32E+06]	***
	Recovery	-3.79E+04 [-2.32E+06 – 2.93E+06]	1.95E+06 [-5.74E+05 – 4.12E+06]	ns
**Cyclic IMP**	Baseline	-2.18E+05 [-2.00E+06 – 8.26E+06]	1.92E+06 [-6.76E+06 – 1.33E+07]	ns
	Free-Wheel	1.32E+06 [-4.19E+06 – 7.22E+06]	-1.63E+06 [-4.72E+06 – 4.57E+06]	ns
	Peak Exercise	1.30E+06 [-4.88E+06 – 5.20E+06]	5.99E+05 [-5.12E+06 – 6.57E+06]	ns
	Recovery	-1.28E+06 [-6.11E+06 – 1.74E+06]	3.82E+06 [-2.55E+06 – 9.28E+06]	****
**Lactate**	Baseline	-1.24E+05 [-9.33E+05 – 4.36E+05]	7.81E+05 [-9.21E+03 – 2.15E+06]	ns
	Free-Wheel	-7.97E+05 [-2.49E+06 – 8.74E+05]	7.74E+05 [-4.65E+05 – 2.22E+06]	ns
	Peak Exercise	-1.24E+06 [-5.87E+06 – 2.02E+06]	2.33E+06 [-2.10E+06 – 9.10E+06]	****
	Recovery	-1.49E+06 [-5.02E+06 – 4.00E+06]	-1.24E+06 [-8.55E+06 – 4.52E+06]	ns
**FA(16:0)**	Baseline	7.62E+04 [-2.07E+07 – 9.03E+06]	7.82E+06 [-6.87E+06 – 2.47E+07]	****
	Free-Wheel	-6.13E+05 [-1.33E+07 – 9.24E+06]	-1.58E+06 [-8.09E+06 – 1.01E+07]	ns
	Peak Exercise	9.17E+05 [-6.85E+06 – 1.24E+07]	3.80E+06 [-1.43E+05 – 1.55E+07]	**
	Recovery	-2.00E+06 [-1.26E+07 – 9.55E+06]	2.04E+06 [-1.73E+07 – 1.37E+07]	ns
**FA(18:1)**	Baseline	3.56E+06 [-1.39E+07 – 1.92E+07]	-1.09E+06 [-1.87E+07 – 1.87E+07]	ns
	Free-Wheel	-3.28E+06 [-2.27E+07 – 1.15E+07]	5.55E+06 [-9.08E+06 – 2.68E+07]	****
	Peak Exercise	8.22E+05 [-7.12E+06 – 1.20E+07]	5.03E+06 [-7.87E+05 – 2.29E+07]	****
	Recovery	1.10E+06 [-1.21E+07 – 1.63E+07]	1.02E+07 [-2.30E+07 – 2.71E+07]	*

Supplemental Table 9

		Black	White	Mixed or Other	Adjusted P Value
**Phenylalanine**	Baseline	1.92E+06 [-2.60E+06 – 2.81E+06]	8.70E+05 [-2.84E+06 – 2.09E+06]	8.89E+05 [-5.94E+05 – 1.96E+06]	ns
	Free-Wheel	3.47E+06 [-1.28E+06 – 5.73E+06]	6.16E+05 [-3.07E+06 – 2.54E+06]	1.32E+06 [-1.91E+06 – 4.17E+06]	ns
	Peak Exercise	-3.34E+06 [-5.07E+06 – -8.98E+05]	8.74E+05 [-1.01E+06 – 3.93E+06]	-9.13E+05 [-2.58E+06 – 1.21E+06]	****, §§§§
	Recovery	6.55E+05 [-3.65E+06 – 3.29E+06]	-9.97E+03 [-2.12E+06 – 3.66E+06]	2.22E+05 [-1.42E+06 – 3.46E+06]	ns
**Cyclic IMP**	Baseline	-7.11E+05 [-2.96E+06 – 1.51E+07]	-1.18E+05 [-2.16E+06 – 7.32E+06]	1.92E+06 [-1.90E+06 – 1.24E+07]	*, §
	Free-Wheel	3.32E+06 [1.79E+06 – 8.33E+06]	-8.39E+05 [-3.39E+06 – 6.65E+06]	1.50E+06 [-8.39E+06 – 7.43E+06]	†
	Peak Exercise	-3.98E+06 [-5.86E+06 – 1.95E+06]	2.66E+06 [-4.01E+06 – 5.68E+06]	6.62E+05 [-6.88E+06 – 5.35E+06]	**, §§
	Recovery	1.97E+05 [-6.14E+06 – 7.39E+06]	-2.33E+06 [-6.13E+06 – 1.89E+06]	4.31E+05 [-4.68E+06 – 4.70E+06]	††
**Lactate**	Baseline	-8.40E+05 [-1.17E+06 – -1.01E+05]	2.22E+05 [-2.89E+05 – 1.44E+06]	1.55E+05 [-6.93E+05 – 1.22E+06]	ns
	Free-Wheel	-7.97E+05 [-1.95E+06 – 1.57E+06]	3.48E+05 [-2.86E+06 – 1.21E+06]	-1.03E+06 [-2.11E+06 – 9.53E+05]	ns
	Peak Exercise	-4.70E+06 [-6.25E+06 – -6.21E+05]	9.71E+05 [-6.97E+06 – 2.87E+06]	1.84E+06 [-2.23E+06 – 4.05E+06]	*, ††††, §§§§
	Recovery	-4.35E+06 [-5.37E+06 – -2.53E+06]	-1.10E+06 [-6.19E+06 – 5.29E+06]	8.60E+05 [-2.95E+06 – 6.36E+06]	*, †††
**Carnitine**	Baseline	2.52E+05 [-3.62E+06 – 1.76E+07]	4.43E+05 [-1.31E+06 – 2.23E+06]	-8.32E+04 [-1.78E+06 – 2.08E+06]	*, †
	Free-Wheel	1.59E+06 [1.79E+05 – 3.20E+06]	-2.06E+05 [-1.34E+06 – 1.28E+06]	-6.21E+04 [-2.50E+06 – 9.41E+05]	ns
	Peak Exercise	-8.75E+05 [-2.76E+06 – 8.19E+05]	4.33E+05 [-1.84E+06 – 2.74E+06]	-6.14E+05 [-1.82E+06 – 9.46E+05]	ns
	Recovery	-2.43E+05 [-6.36E+05 – 1.50E+06]	4.07E+05 [-7.17E+05 – 1.62E+06]	-4.55E+05 [-2.28E+06 – 1.39E+06]	ns
**FA(16:0)**	Baseline	6.21E+06 [-2.91E+07 – 1.35E+07]	1.81E+06 [-1.02E+07 – 1.00E+07]	-3.72E+06 [-2.03E+07 – 5.91E+06]	****, §§§§
	Free-Wheel	1.20E+07 [-1.69E+07 – 1.82E+07]	-1.93E+06 [-2.26E+07 – 1.14E+07]	-2.10E+06 [-1.10E+07 – 3.64E+06]	†
	Peak Exercise	-6.30E+06 [-1.23E+07 – 7.34E+05]	6.16E+06 [-4.48E+06 – 1.81E+07]	1.63E+06 [-5.00E+06 – 1.06E+07]	****, ††††, §§
	Recovery	-7.17E+06 [-2.98E+07 – 2.48E+05]	4.22E+06 [-8.33E+06 – 9.97E+06]	-2.33E+06 [-1.63E+07 – 1.32E+07]	****, ††††
**FA(18:0)**	Baseline	1.25E+06 [-2.87E+06 – 7.32E+06]	1.11E+06 [-4.71E+06 – 6.06E+06]	1.92E+05 [-4.19E+06 – 4.45E+06]	†, §§
	Free-Wheel	5.65E+06 [-8.64E+06 – 6.22E+06]	-2.56E+05 [-3.86E+06 – 3.38E+06]	-1.36E+06 [-6.38E+06 – 5.03E+06]	ns
	Peak Exercise	5.77E+05 [-6.57E+06 – 5.63E+06]	3.59E+06 [-8.89E+05 – 8.30E+06]	4.77E+05 [-3.03E+06 – 3.64E+06]	**, §§
	Recovery	-3.47E+06 [-4.13E+06 – 2.09E+06]	-2.96E+05 [-4.76E+06 – 4.47E+06]	7.96E+05 [-2.38E+06 – 3.00E+06]	ns
**FA(18:1)**	Baseline	-1.32E+07 [-2.45E+07 – 1.64E+07]	4.36E+06 [-1.28E+07 – 2.02E+07]	9.88E+04 [-1.43E+07 – 1.89E+07]	****, ††††
	Free-Wheel	9.55E+06 [-2.52E+07 – 2.53E+07]	-1.04E+07 [-2.40E+07 – 1.53E+06]	9.14E+06 [-3.67E+06 – 1.97E+07]	****, †, §§§§
	Peak Exercise	-4.82E+06 [-1.85E+07 – 2.08E+05]	6.50E+06 [-3.02E+06 – 2.82E+07]	8.18E+05 [-1.09E+07 – 6.80E+06]	****, ††††, §§§§
	Recovery	-3.28E+07 [-5.41E+07 – 2.25E+07]	1.12E+06 [-1.55E+07 – 1.77E+07]	9.59E+06 [-3.24E+06 – 2.61E+07]	****, ††††, §§§§

Supplemental Table 10

Metric	Value
**AUC**	0.85327
**Accuracy**	0.80543
**Sensitivity**	0.95172
**Specificity**	0.52632

Supplemental Figure 1

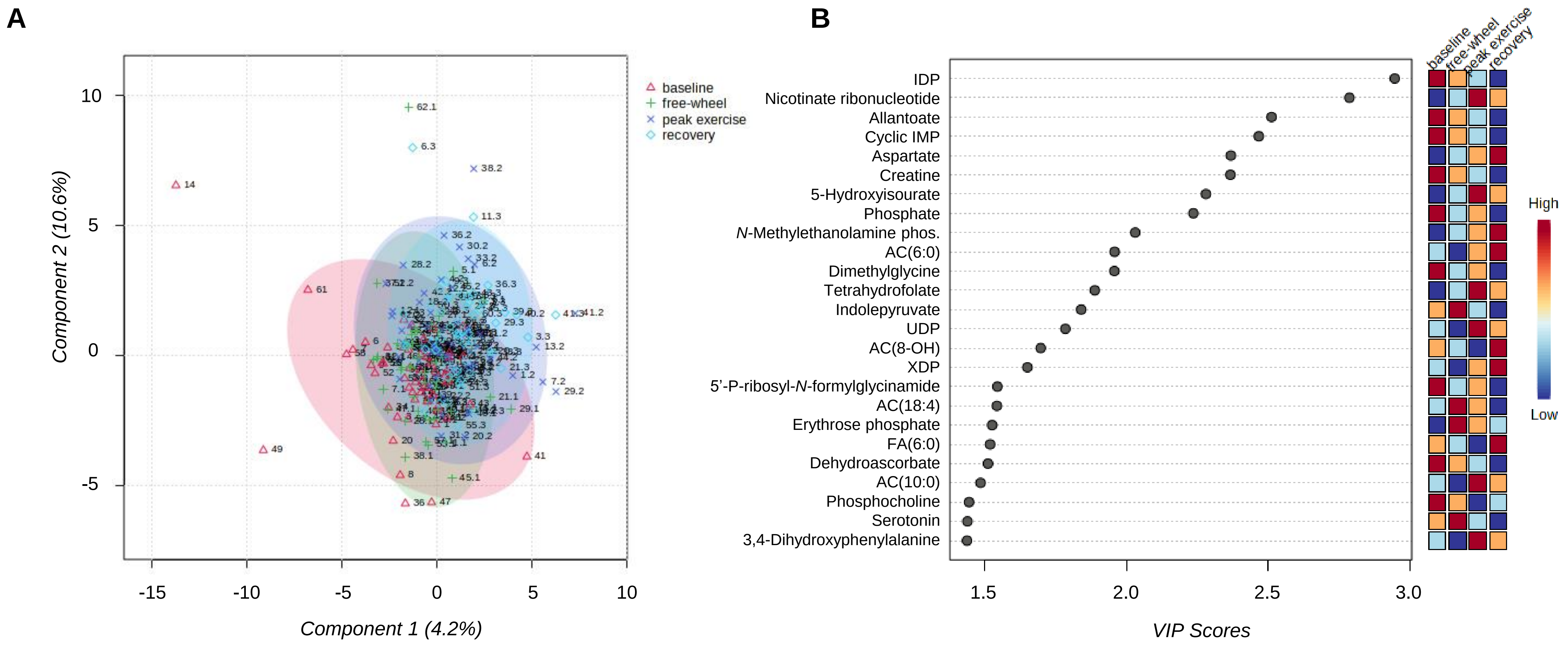

Supplemental Figure 2

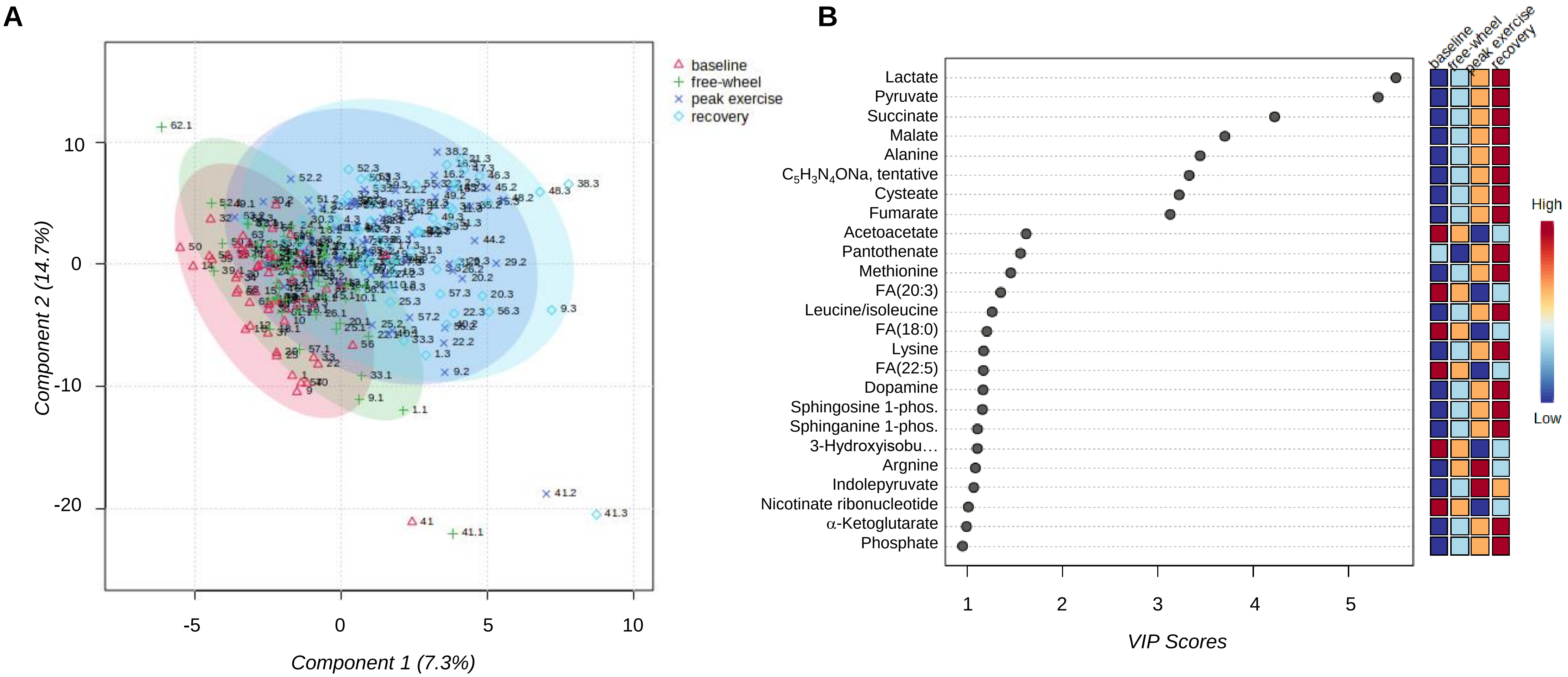

Supplemental Figure 3

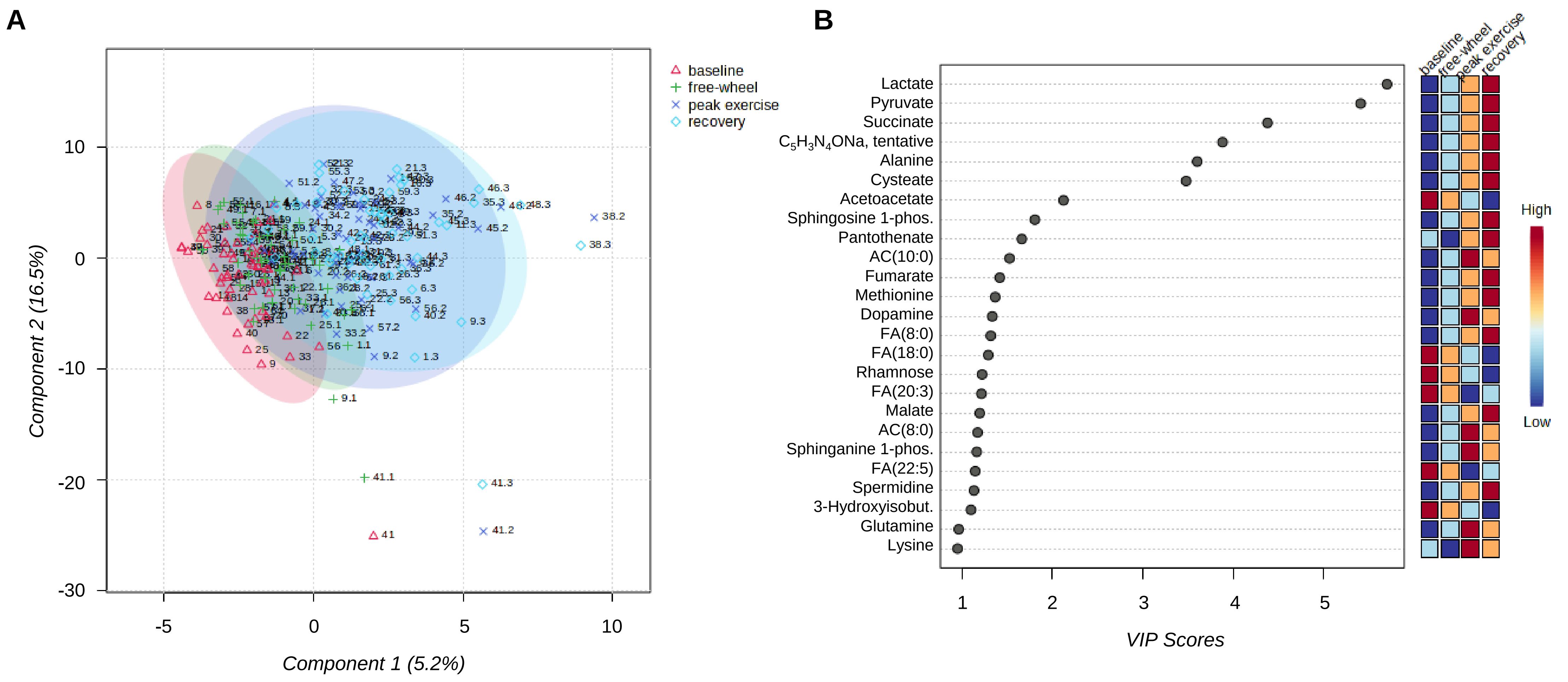

Supplemental Figure 4

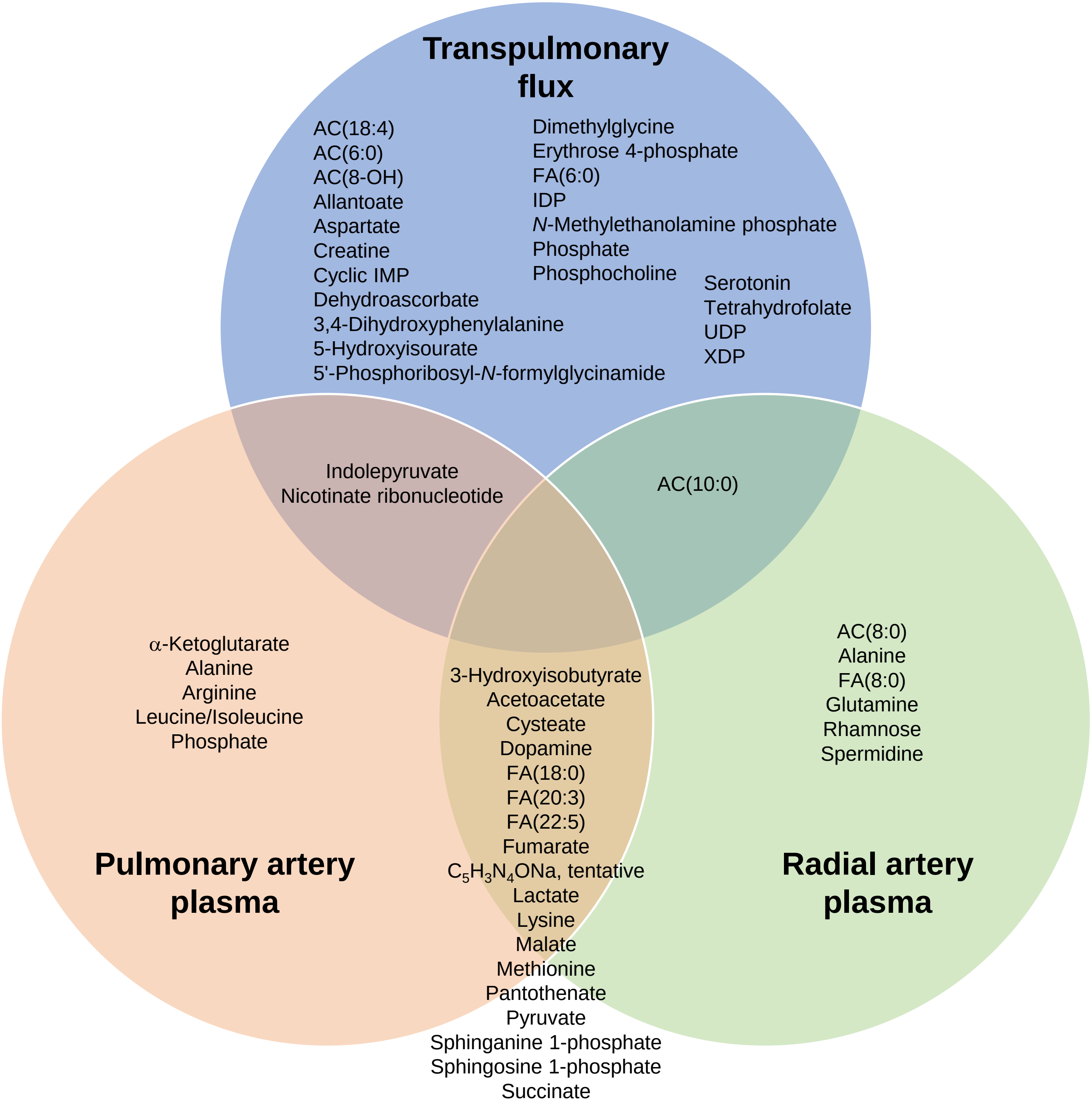

Supplemental Figure 5

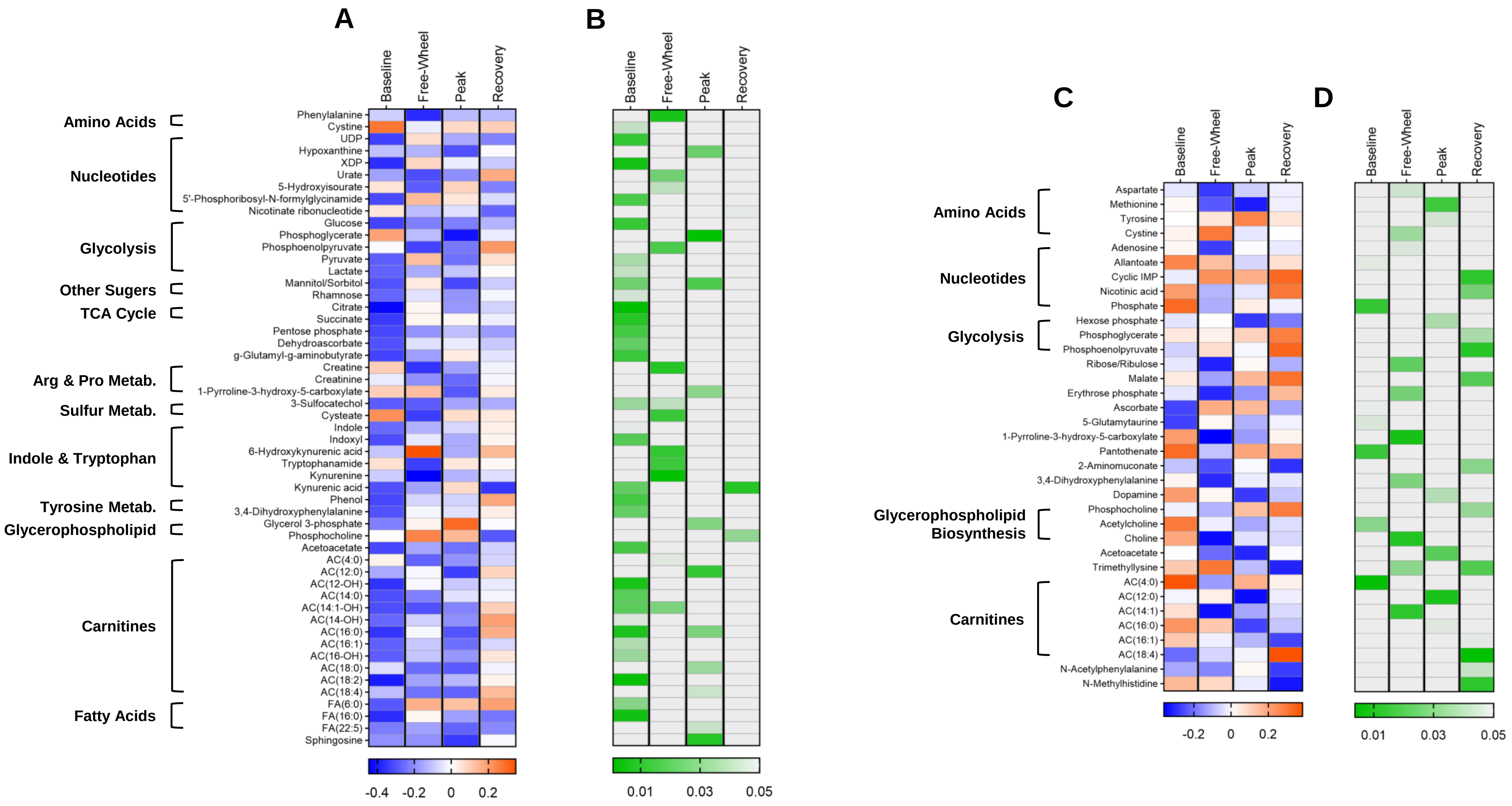

Supplemental Figure 6

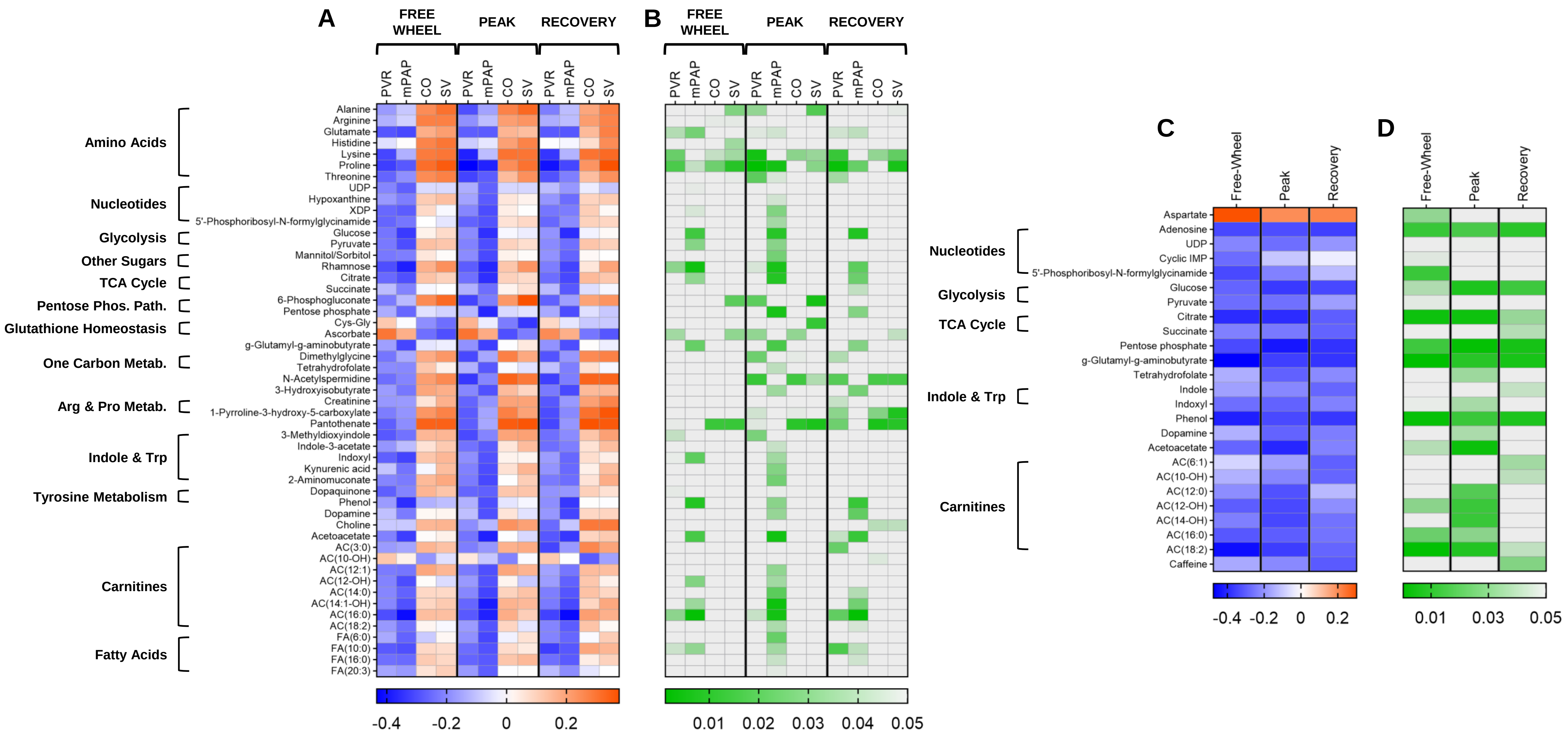

Supplemental Figure 7

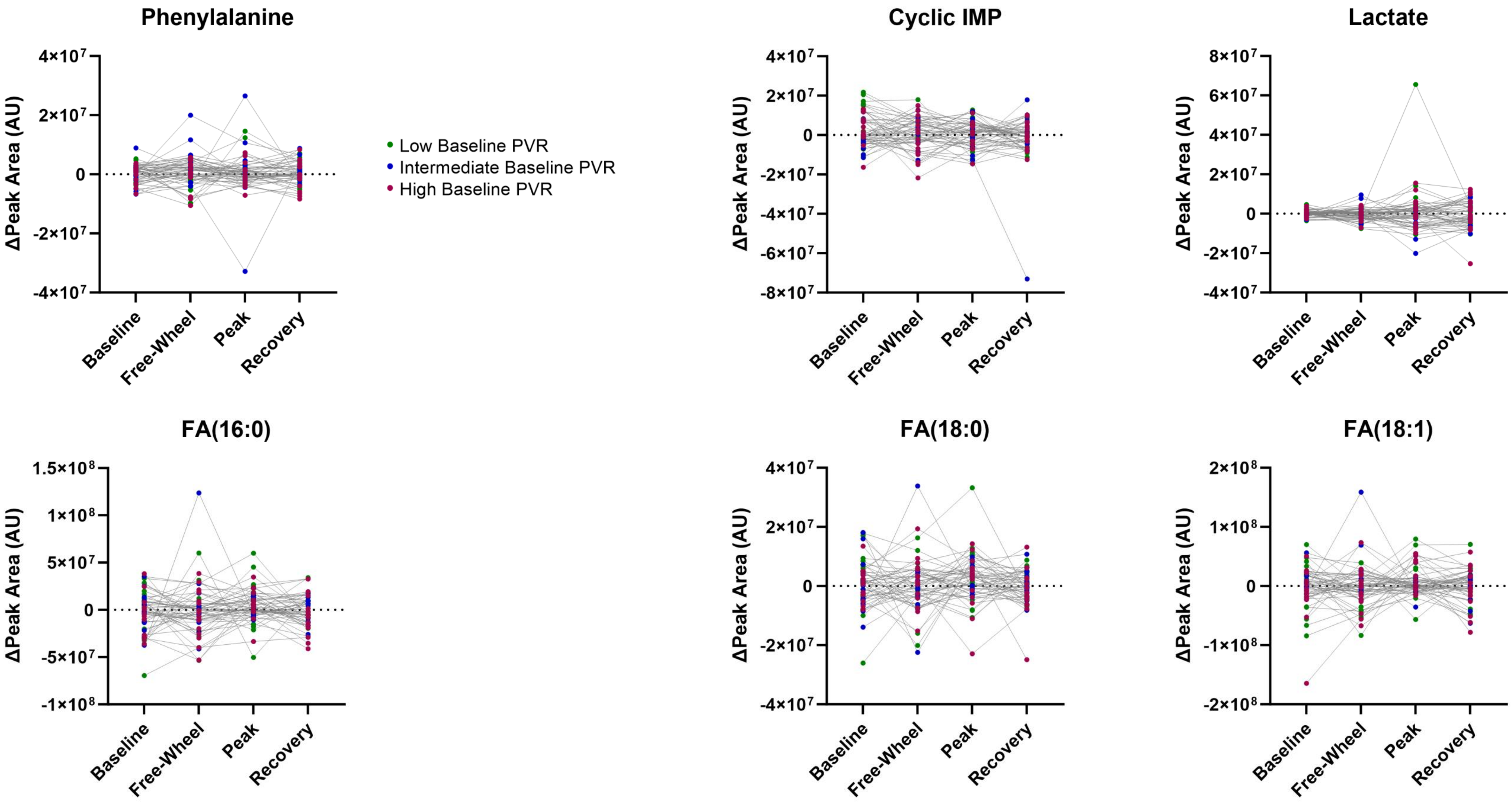

Supplemental Figure 8

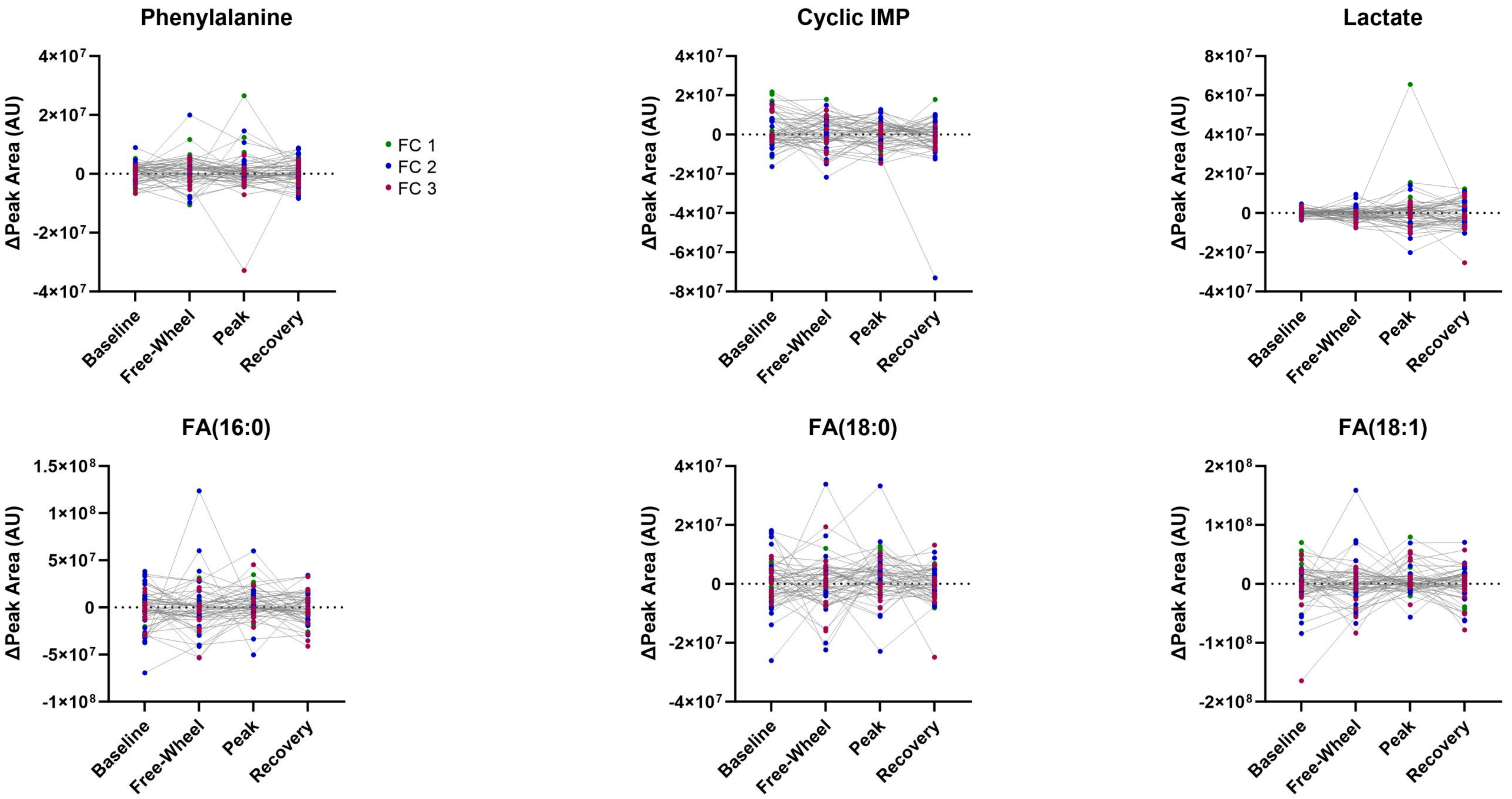

Supplemental Figure 9

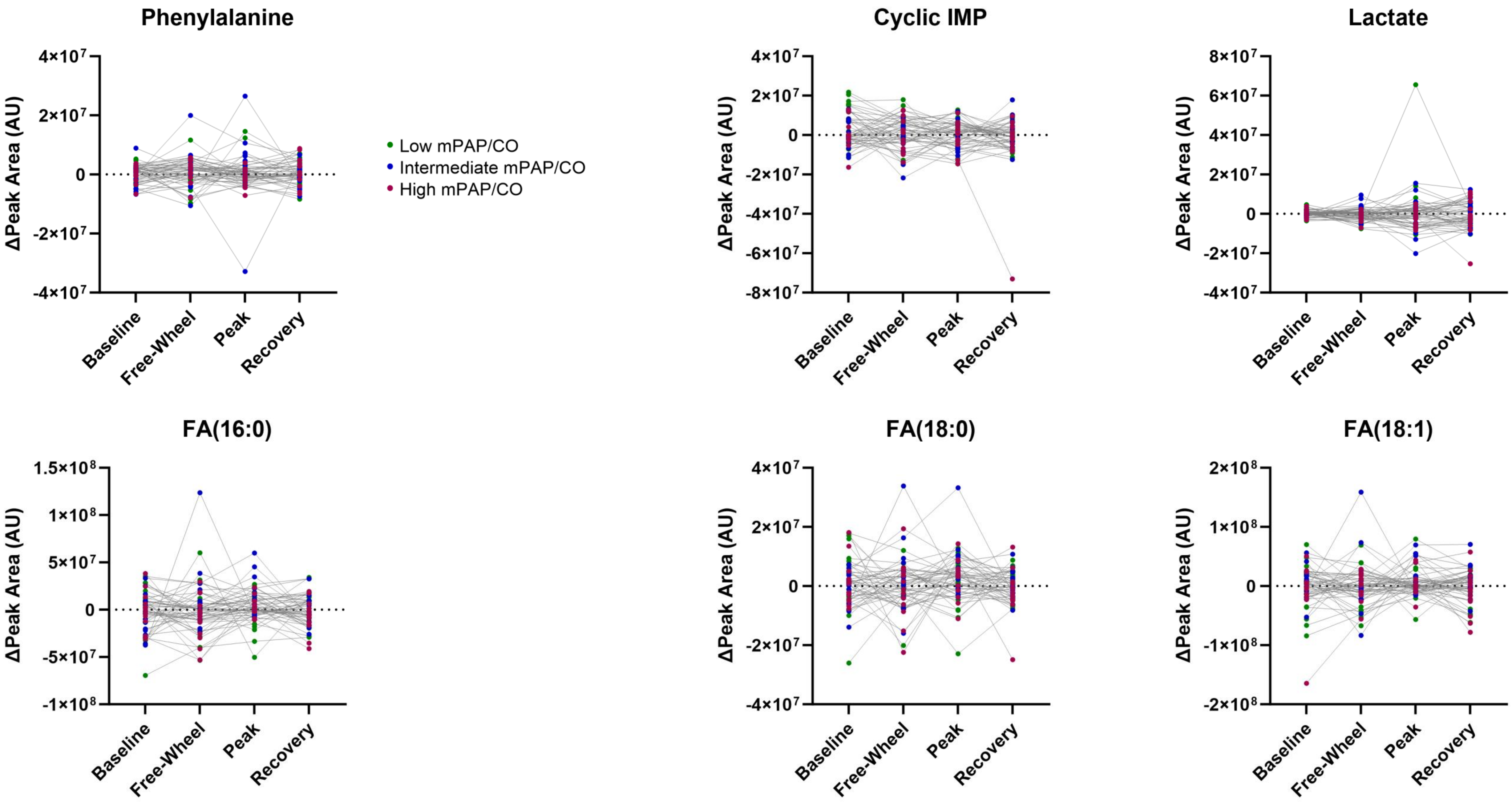

Supplemental Figure 10

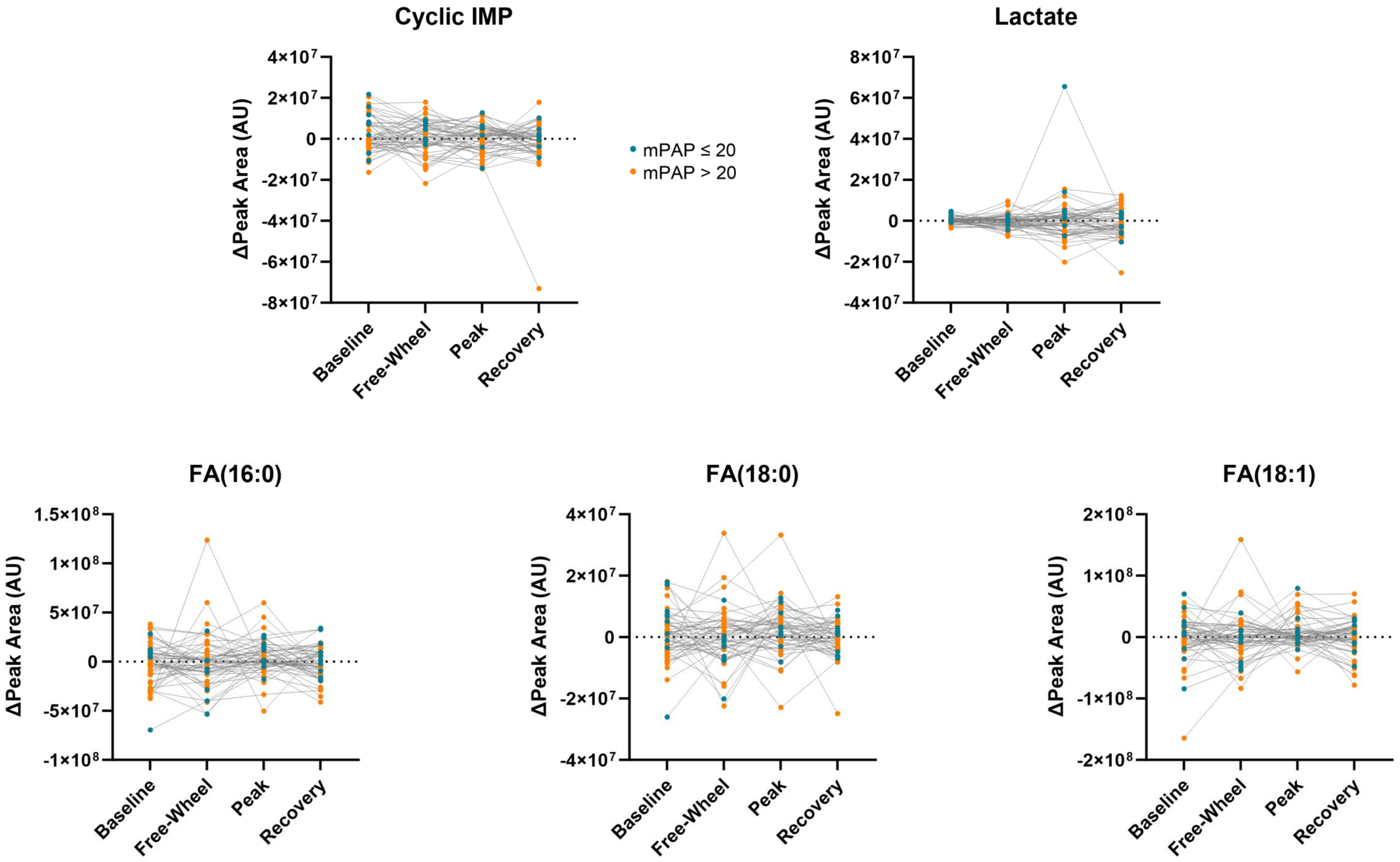

Supplemental Figure 11

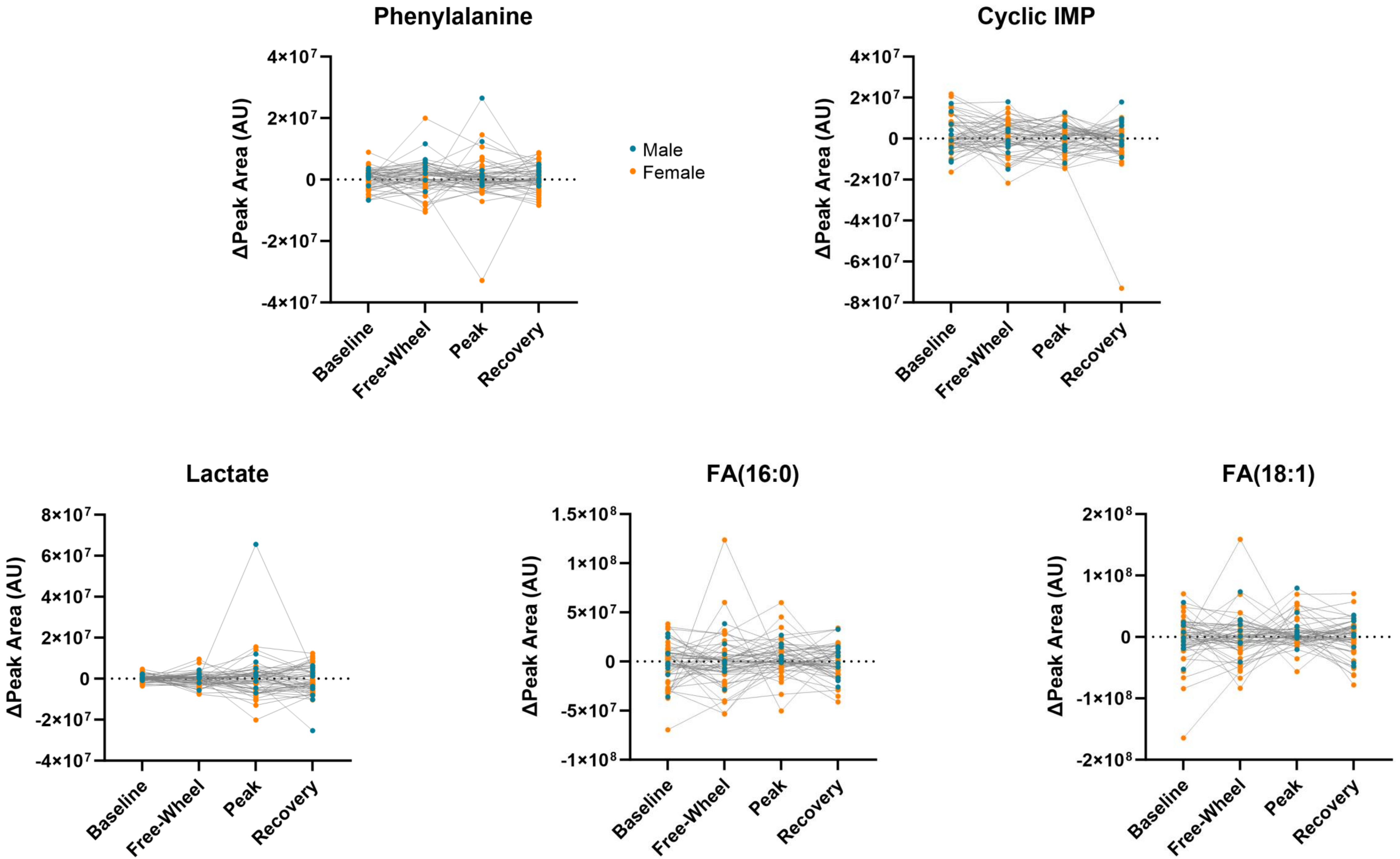

Supplemental Figure 12

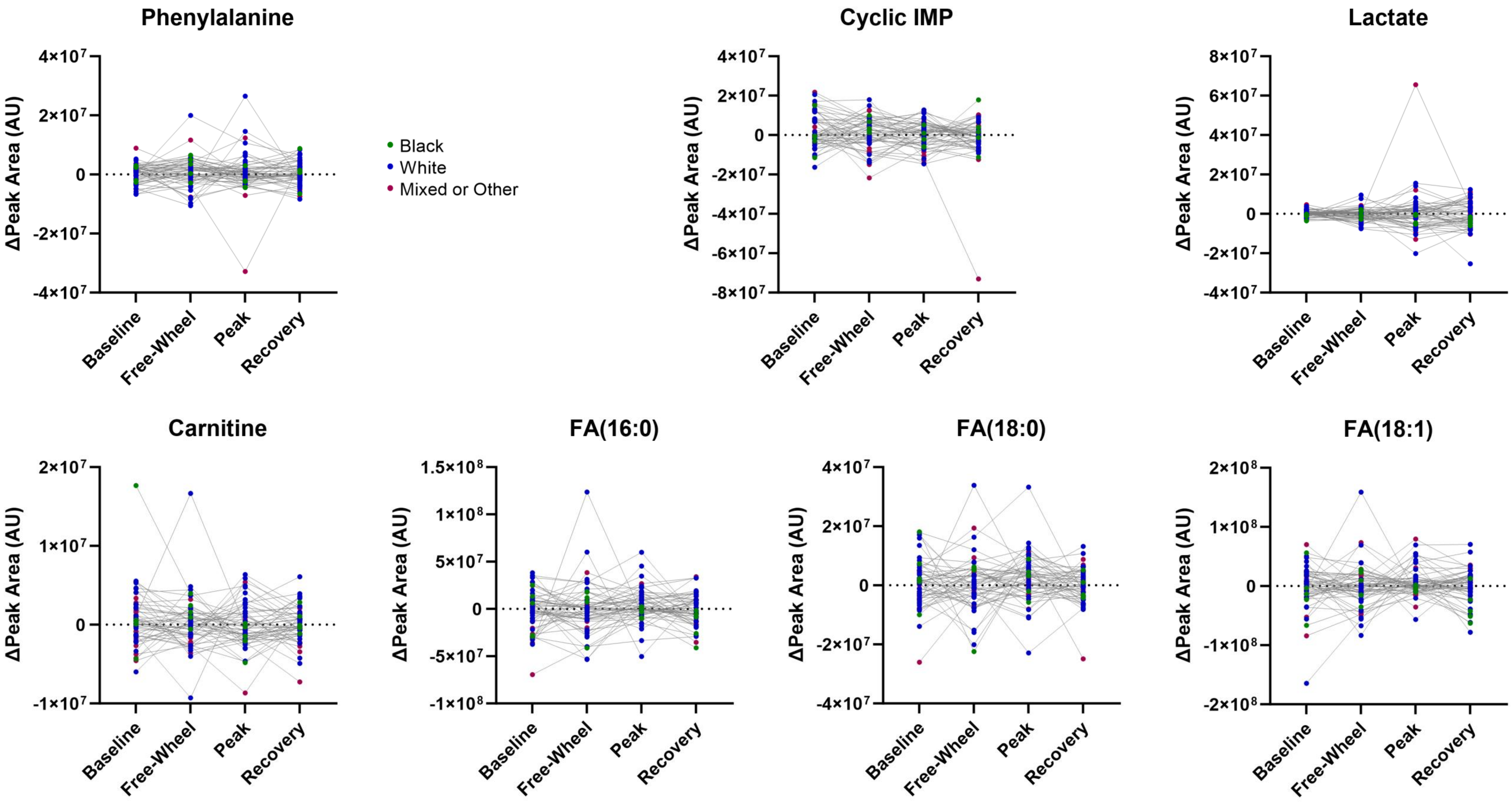

Supplemental Table and Figure Legends

Supplemental Table 1. Hemodynamic indices during the four stages of exercise. Descriptive statistics of individual values are expressed in median [interquartile range] (n = 63 for baseline, 59 for free-wheel, 62 for peak exercise, and 62 for recovery). Adjusted p values represent repeated measures statistics computed using Tukey's multiple comparisons test on mixed-effects model with n = 59/group. The p value symbols denote the following comparisons: baseline vs. free-wheel (*), peak exercise (†), or recovery (‡); free-wheel vs. peak exercise (§) or recovery (#); peak exercise vs. recovery (¶). The number of these symbols, from one to four, denote $p < 0.05$, $p < 0.01$, $p < 0.001$, and $p < 0.0001$, respectively (e.g., ¶¶¶¶ indicates $p < 0.0001$ between peak exercise vs. recovery). Definition of abbreviations: BP = blood pressure; CI = cardiac index; CO = cardiac output; MAP = systemic mean arterial pressure; mPAP = mean pulmonary artery pressure; PA = pulmonary artery; PAC = pulmonary arterial compliance; PVR = pulmonary vascular resistance; PVRi = pulmonary vascular resistance index; RAP = right atrial pressure; RVSWI = right ventricular stroke work index; SaO2 = systemic arterial oxygen saturation; SmvO2 = pulmonary mixed-venous oxygen saturation; SV = stroke volume; SVi = stroke volume index; SVR = systemic vascular resistance; SVRi = systemic vascular resistance index; TD = thermodilution.

Supplemental Table 2. The listed 32 stable isotope-labeled metabolites of known concentrations were added and measured in all plasma samples of the 63 participants, from both anatomic sites when feasible and during all available exercise stages. Using simple linear regression, each isotope-labeled metabolite was correlated with the semi-

quantitative peak area of the same metabolite to ensure the accuracy and reliability of our mass spectrometric measurements. All 32 metabolites demonstrated significant ($p < 0.0001$) and positive correlations. Definition of abbreviations: AC = acylcarnitine; CmpdID = compound identification; FA = fatty acid; TCA = tricarboxylic acid.

Supplemental Table 3. Effect size calculations for the pulmonary artery vs. radial artery comparisons of individual metabolites (Figure 3). For the effect size of each metabolite, the absolute difference between the pulmonary artery and radial artery mean values was divided by their pooled standard deviation. Definition of abbreviations: IDP = inosine diphosphate; PA = pulmonary artery; Rad = radial artery; SD = standard deviation.

Supplemental Table 4. Subgroup analysis of transpulmonary metabolite flux according to baseline PVR. Descriptive statistics of individual values are expressed in median [interquartile range]. Number of subjects (n) of the low/intermediate/high PVR groups are: 19/19/19 for Baseline; 19/17/17 for Free-Wheel; 19/19/18 for Peak Exercise and Recovery, respectively. The p value symbols denote the following comparisons: low vs. intermediate baseline PVR (*); low vs. high baseline PVR (†), or intermediate vs. high baseline PVR (§). The number of the symbols, from one to four, denote $p < 0.05$, $p < 0.01$, $p < 0.001$, and $p < 0.0001$, respectively (e.g., **** indicates $p < 0.0001$). Statistical analyses were performed using ordinary Two-way ANOVA with multiple comparisons corrected with the Tukey method. Definition of abbreviations: Cyclic IMP = inosine cyclic 3',5' monophosphate; FA = fatty acid; PVR = pulmonary vascular resistance.

Supplemental Table 5. Subgroup analysis of transpulmonary metabolite flux according to functional class. Descriptive statistics of individual values are expressed in median [interquartile range]. Number of subjects (n) of the FC I/II/III groups are: 13/27/17 for Baseline; 13/26/14 for Free-Wheel; 13/27/16 for Peak Exercise and Recovery, respectively. The p value symbols indicate: FC 1 vs. 2 (*); 1 vs. 3 (†), or 2 vs. 3 (§). The number of the symbols, from one to four, denote $p < 0.05$, $p < 0.01$, $p < 0.001$, and $p < 0.0001$, respectively (e.g., **** indicates $p < 0.0001$). Statistical analyses were performed using ordinary Two-way ANOVA with multiple comparisons corrected with the Tukey method. Definition of abbreviations: Cyclic IMP = inosine cyclic 3',5' monophosphate; FA = fatty acid; FC = functional class.

Supplemental Table 6. Subgroup analysis of transpulmonary metabolite flux according to mPAP/CO. Descriptive statistics of individual values are expressed in median [interquartile range]. Number of subjects (n) of the low/intermediate/high mPAP/CO groups are: 19/19/18 for Baseline; 19/19/15 for Free-Wheel; 19/19/18 for Peak Exercise and Recovery, respectively. The p value symbols indicate the following comparisons: low vs. intermediate mPAP/CO (*); low vs. high mPAP/CO (†), or intermediate vs. high mPAP/CO (§). The number of the symbols, from one to four, denote $p < 0.05$, $p < 0.01$, $p < 0.001$, and $p < 0.0001$, respectively (e.g., **** indicates $p < 0.0001$). Statistical analyses were performed using ordinary Two-way ANOVA with multiple comparisons corrected with the Tukey method. Definition of abbreviations: Cyclic IMP = inosine cyclic

3',5' monophosphate; FA = fatty acid; mPAP/CO = mean pulmonary artery pressure to cardiac output ratio.

Supplemental Table 7. Subgroup analysis of transpulmonary metabolite flux according to baseline mean pulmonary arterial pressure (mPAP), either non-elevated (≤ 20) or elevated (>20). Descriptive statistics of individual values are expressed in median [interquartile range]. Number of subjects (n) of the non-elevated/elevated baseline mPAP groups are: 10/47 for Baseline; 10/43 for Free-Wheel; 10/46 for Peak Exercise and Recovery, respectively. The number of the * symbol, from one to four, denote $p < 0.05$, $p < 0.01$, $p < 0.001$, and $p < 0.0001$, respectively (e.g., **** indicates $p < 0.0001$). Statistical analyses were performed using ordinary Two-way ANOVA with multiple comparisons corrected with the Šidák method. Definition of abbreviations: Cyclic IMP = inosine cyclic 3',5' monophosphate; FA = fatty acid.

Supplemental Table 8. Subgroup analysis of transpulmonary metabolite flux according to sex. Descriptive statistics of individual values are expressed in median [interquartile range]. Number of subjects (n) of female/male groups are: 46/11 for Baseline; 43/10 for Free-Wheel; 46/10 for Peak Exercise and Recovery, respectively. The number of the * symbol, from one to four, denote $p < 0.05$, $p < 0.01$, $p < 0.001$, and $p < 0.0001$, respectively (e.g., **** indicates $p < 0.0001$). Statistical analyses were performed using ordinary Two-way ANOVA with multiple comparisons corrected with the Šidák method. Definition of abbreviations: Cyclic IMP = inosine cyclic 3',5' monophosphate; FA = fatty acid.

Supplemental Table 9. Subgroup analysis of transpulmonary metabolite flux according to race or ethnic group. Descriptive statistics of individual values are expressed in median [interquartile range]. Number of subjects (n) of the black/white/mixed or other groups are: 7/31/19 for Baseline; 5/30/18 for Free-Wheel; 6/31/19 for Peak Exercise and Recovery, respectively. The p value symbols denote the following comparisons: black vs. white (*); black vs. mixed or other (†), or white vs. mixed or other (§). The number of the symbols, from one to four, denote $p < 0.05$, $p < 0.01$, $p < 0.001$, and $p < 0.0001$, respectively (e.g., **** indicates $p < 0.0001$). Statistical analyses were performed using ordinary Two-way ANOVA with multiple comparisons corrected with the Tukey method. Definition of abbreviations: Cyclic IMP = inosine cyclic 3',5' monophosphate; FA = fatty acid.

Supplemental Table 10. Performance metrics of the LASSO (least absolute shrinkage and selection operator) regression analysis in Figure 6. Definition of abbreviation: AUC = area under the curve.

Supplemental Figure 1. Differentiation of the four exercise stages in transpulmonary plasma samples. **(A)**. A partial least squares-discriminant analysis (PLS-DA) plot of transpulmonary (radial artery – pulmonary artery) metabolite flux shows separations and significant overlaps among the four exercise stages. **(B)**. A variable importance in projection (VIP) score plot shows 25 metabolites; transpulmonary gradients of these metabolites are associated with top VIP scores, indicative of highest discriminating

power amongst the four exercise stages. Metabolite peak areas were autoscaled using MetaboAnalyst v 6.0. Definition of abbreviations: AC = acylcarnitine; FA = fatty acid. Definition of abbreviations: AC = acylcarnitine; Cyclic IMP = inosine cyclic 3',5' monophosphate; FA = fatty acid; IDP = inosine diphosphate; UDP = uridine diphosphate; XDP = xanthosine diphosphate.

Supplemental Figure 2. Differentiation of the four exercise stages in pulmonary artery plasma samples. **(A)**. A PLS-DA plot of pulmonary artery metabolites shows a separation of the four stages with a chronological layout. For example, metabolites of the free-wheel stage in green are positioned between those of the baseline and the peak exercise stages. **(B)**. A variable importance in projection (VIP) score plot shows 25 pulmonary artery metabolites with top VIP scores, indicative of highest discriminating power amongst the four exercise stages. Metabolite peak areas were autoscaled using MetaboAnalyst v 6.0. Definition of abbreviation: FA = fatty acid.

Supplemental Figure 3. Differentiation of the four exercise stages in radial artery plasma samples. **(A)**. A PLS-DA plot of radial artery metabolites shows a separation of the four stages with a chronological layout. For example, metabolites of the free-wheel stage in green are positioned between those of the baseline and the peak exercise stages. **(B)**. A VIP score plot shows 25 radial artery metabolites with top VIP scores, indicative of highest discriminating power amongst the four exercise stages. Metabolite peak areas were autoscaled using MetaboAnalyst v 6.0. Definition of abbreviations: AC = acylcarnitine; FA = fatty acid.

Supplemental Figure 4. Transpulmonary (radial – pulmonary artery) analysis reveals a unique set of metabolites differentiating the four exercise stages. Metabolites with top 25 VIP scores are listed in groups, based on plasma collection site. Plasma samples from the pulmonary artery (PA) and the systemic radial artery (Rad) share multiple common metabolites, but the overlap is minimal in comparison to the transpulmonary (Rad-PA) values. Definition of abbreviations: AC = acylcarnitine; Cyclic IMP = inosine cyclic 3',5' monophosphate; FA = fatty acid; IDP = inosine diphosphate; UDP = uridine diphosphate; XDP = xanthosine diphosphate.

Supplemental Figure 5. A-B. Correlations between right ventricular stroke work index (RVSWI) and transpulmonary metabolite flux within each exercise stage. Spearman correlation coefficients are shown on the left (A), and p values are shown on the right (B). For example, peak exercise ("PEAK") statistics represent correlations between RVSWI and metabolites both measured during peak exercise. C-D. Correlations between maximum work rate (Watts) generated at peak exercise and transpulmonary metabolite flux at each of the four exercise stages. Spearman correlation coefficients are shown on the left (C), and p values are shown on the right (D). Spearman rank correlations were performed using MetaboAnalyst v 6.0, and only significant metabolites ($p < 0.05$ in at least one exercise stage) are included in the heatmaps. Definition of abbreviations: AC = acylcarnitine; Arg = arginine; Cyclic IMP = inosine cyclic 3',5' monophosphate; FA = fatty acid; Pro = proline; TCA = tricarboxylic acid; UDP = uridine diphosphate; XDP = xanthosine diphosphate.

Supplemental Figure 6. A-B. Correlations between baseline transpulmonary metabolite flux and hemodynamic indices measured during the other three exercise stages. Spearman correlation coefficients are shown on the left (A), and p values are shown on the right (B). For example, peak exercise (“PEAK”) statistics represent correlations between hemodynamic variables measured during peak exercise and metabolites measured during rest (baseline). C-D. Correlations between baseline transpulmonary metabolite flux and right ventricular stroke work index (RVSWI) measured during the other three exercise stages. Spearman correlation coefficients are shown on the left (C), and p values are shown on the right (D). Spearman rank correlations were performed using MetaboAnalyst v 6.0, and only significant metabolites ($p < 0.05$ in at least one exercise stage) are included in the heatmaps. Definition of abbreviations: AC = acylcarnitine; Arg = arginine; Cys-Gly = cysteinylglycine; Cyclic IMP = inosine cyclic 3',5’ monophosphate; FA = fatty acid; Pro = proline; TCA = tricarboxylic acid; Trp = tryptophan; UDP = uridine diphosphate; XDP = xanthosine diphosphate.

Supplemental Figure 7. Graphical illustrations of transpulmonary metabolite flux in the three subgroups according to baseline PVR (Supplemental Table 3). Demonstrated are individual gradeints of the 53 participants who completed all four stages of exercise. Definition of abbreviations: AU = arbitrary unit; Cyclic IMP = inosine cyclic 3',5’ monophosphate; FA = fatty acid; PVR = pulmonary vascular resistance.

Supplemental Figure 8. Graphical illustrations of transpulmonary metabolite flux in the three subgroups according to functional class (FC) (Supplemental Table 4). Demonstrated are individual gradeints of the 53 participants who completed all four stages of exercise. Definition of abbreviations: AU = arbitrary unit; Cyclic IMP = inosine cyclic 3',5' monophosphate; FA = fatty acid; FC = functional class.

Supplemental Figure 9. Graphical illustrations of transpulmonary metabolite flux in the three subgroups according to mean pulmonary artery pressure (mPAP) to cardiac output (CO) ratio (mPAP/CO) (Supplemental Table 5). Demonstrated are individual gradeints of the 53 participants who completed all four stages of exercise. Definition of abbreviations: AU = arbitrary unit; Cyclic IMP = inosine cyclic 3',5' monophosphate; FA = fatty acid.

Supplemental Figure 10. Graphical illustrations of transpulmonary metabolite flux in the two subgroups according to baseline mean pulmonary artery pressure (mPAP) (Supplemental Table 6). Demonstrated are individual gradeints of the 53 participants who completed all four stages of exercise. Definition of abbreviations: AU = arbitrary unit; Cyclic IMP = inosine cyclic 3',5' monophosphate; FA = fatty acid.

Supplemental Figure 11. Graphical illustrations of transpulmonary metabolite flux in the two subgroups according to sex (Supplemental Table 7). Demonstrated are individual gradeints of the 53 participants who completed all four stages of exercise.

Definition of abbreviations: AU = arbitrary unit; Cyclic IMP = inosine cyclic 3',5’ monophosphate; FA = fatty acid.

Supplemental Figure 12. Graphical illustrations of transpulmonary metabolite flux in the three subgroups according to race or ethnic group (Supplemental Table 8). Demonstrated are individual gradeints of the 53 participants who completed all four stages of exercise. Definition of abbreviations: AU = arbitrary unit; Cyclic IMP = inosine cyclic 3',5’ monophosphate; FA = fatty acid.
